# Detection of oxaliplatin- and cisplatin-DNA lesions requires different global genome repair mechanisms that affect their clinical efficacy

**DOI:** 10.1093/narcan/zcad057

**Published:** 2023-12-05

**Authors:** Jana Slyskova, Alba Muniesa-Vargas, Israel Tojal da Silva, Rodrigo Drummond, Jiyeong Park, David Häckes, Isabella Poetsch, Cristina Ribeiro-Silva, Amandine Moretton, Petra Heffeter, Orlando D Schärer, Wim Vermeulen, Hannes Lans, Joanna I Loizou

**Affiliations:** Center for Cancer Research, Medical University of Vienna, A-1090 Vienna, Austria; CeMM Research Center for Molecular Medicine of the Austrian Academy of Sciences, A-1090 Vienna, Austria; Department of Molecular Genetics, Erasmus MC Cancer Institute, Erasmus University Medical Center, 3015 GD Rotterdam, The Netherlands; Laboratory of Bioinformatics and Computational Biology, A.C. Camargo Cancer Center, São Paulo 01508-010, Brazil; Laboratory of Bioinformatics and Computational Biology, A.C. Camargo Cancer Center, São Paulo 01508-010, Brazil; Center for Genomic Integrity, Institute for Basic Science, Ulsan 44919, Republic of Korea; Department of Molecular Genetics, Erasmus MC Cancer Institute, Erasmus University Medical Center, 3015 GD Rotterdam, The Netherlands; Center for Cancer Research, Medical University of Vienna, A-1090 Vienna, Austria; Research Cluster “Translational Cancer Therapy Research”, A-1090 Vienna, Austria; Department of Molecular Genetics, Erasmus MC Cancer Institute, Erasmus University Medical Center, 3015 GD Rotterdam, The Netherlands; Center for Cancer Research, Medical University of Vienna, A-1090 Vienna, Austria; CeMM Research Center for Molecular Medicine of the Austrian Academy of Sciences, A-1090 Vienna, Austria; Center for Cancer Research, Medical University of Vienna, A-1090 Vienna, Austria; Research Cluster “Translational Cancer Therapy Research”, A-1090 Vienna, Austria; Center for Genomic Integrity, Institute for Basic Science, Ulsan 44919, Republic of Korea; Department of Biological Sciences, Ulsan National Institute of Science and Technology, Ulsan 44919, Republic of Korea; Department of Molecular Genetics, Erasmus MC Cancer Institute, Erasmus University Medical Center, 3015 GD Rotterdam, The Netherlands; Department of Molecular Genetics, Erasmus MC Cancer Institute, Erasmus University Medical Center, 3015 GD Rotterdam, The Netherlands; Center for Cancer Research, Medical University of Vienna, A-1090 Vienna, Austria; CeMM Research Center for Molecular Medicine of the Austrian Academy of Sciences, A-1090 Vienna, Austria

## Abstract

The therapeutic efficacy of cisplatin and oxaliplatin depends on the balance between the DNA damage induction and the DNA damage response of tumor cells. Based on clinical evidence, oxaliplatin is administered to cisplatin-unresponsive cancers, but the underlying molecular causes for this tumor specificity are not clear. Hence, stratification of patients based on DNA repair profiling is not sufficiently utilized for treatment selection. Using a combination of genetic, transcriptomics and imaging approaches, we identified factors that promote global genome nucleotide excision repair (GG-NER) of DNA-platinum adducts induced by oxaliplatin, but not by cisplatin. We show that oxaliplatin-DNA lesions are a poor substrate for GG-NER initiating factor XPC and that DDB2 and HMGA2 are required for efficient binding of XPC to oxaliplatin lesions and subsequent GG-NER initiation. Loss of DDB2 and HMGA2 therefore leads to hypersensitivity to oxaliplatin but not to cisplatin. As a result, low DDB2 levels in different colon cancer cells are associated with GG-NER deficiency and oxaliplatin hypersensitivity. Finally, we show that colon cancer patients with low DDB2 levels have a better prognosis after oxaliplatin treatment than patients with high DDB2 expression. We therefore propose that DDB2 is a promising predictive marker of oxaliplatin treatment efficiency in colon cancer.

## Graphical Abstract

**Graphical Abstract:**
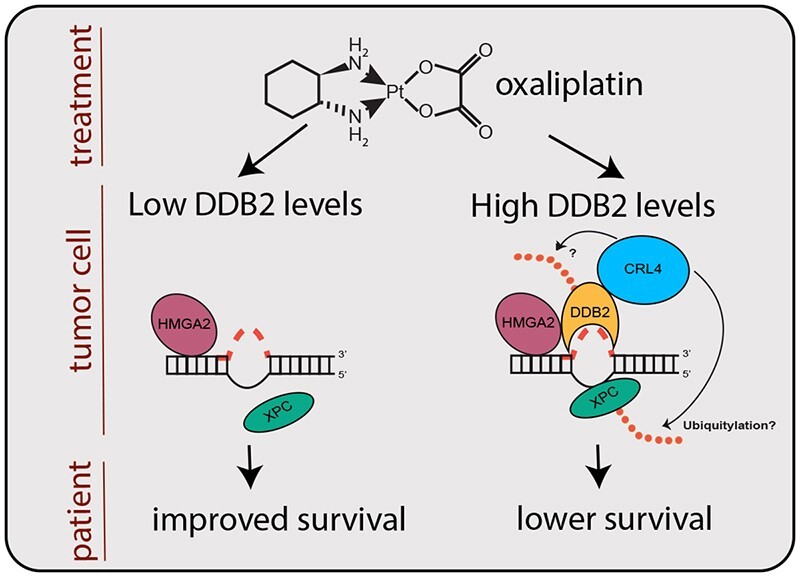

## Introduction

Cisplatin and oxaliplatin are commonly used platinum-based chemotherapeutics that are administered alone or in combination with other drugs to treat a large variety of cancers. Both agents inhibit tumor growth by generating DNA damage that interferes with DNA replication and transcription, thus leading to cell dysfunction and death. Different platinum drugs are used to treat different types of cancer because their responses can vary considerably. Some tumors, like testicular germ cell tumors, are very sensitive to cisplatin treatment, while others, like colorectal tumors, are much more refractory (1–3). Oxaliplatin is therefore used to treat colorectal and other cisplatin-unresponsive types of cancer (4–6). The underlying mechanism for this drug specificity is poorly understood, but it is assumed to be modulated by differences in cellular drug uptake, drug detoxification and defense mechanisms against drug-induced DNA damage (4,7). These defense mechanisms consist of different DNA repair and DNA damage tolerance mechanisms, as well as checkpoint signaling pathways, and are collectively referred to as the DNA damage response (DDR) (8).

Cisplatin and oxaliplatin generate multiple types of platinum-DNA lesions, which are mainly intrastrand crosslinks between adjacent purines as well as interstrand crosslinks (ICLs), monofunctional adducts and DNA-protein crosslinks (7,9,10). Different DNA repair pathways remove these lesions and therefore counteract the effects of chemotherapy. Platinum-ICLs are repaired preferably during replication by a dedicated pathway involving the Fanconi anemia (FA), translesion synthesis (TLS) and homologous recombination (HR) pathways (11). Intrastrand crosslinks and monoadducts are removed by nucleotide excision repair (NER) (12–15). We previously reported that exposure to cisplatin and oxaliplatin also induces oxidative DNA damage, for which repair by base excision repair (BER) is important (16). Using a DDR-dedicated CRISPR/Cas9-based screen, we showed that the above discussed DNA repair pathways are equally important in clearing oxaliplatin-induced DNA damage in DLD-1 colon cancer cells.

NER is a versatile DNA repair pathway that can remove a large range of structurally unrelated helix-distorting DNA lesions via two sub pathways (14,17). Transcription-coupled NER (TC-NER) removes damage from transcribed DNA and is initiated by stable binding of CSB to lesion-stalled RNA polymerase II (18,19). Global genome NER (GG-NER) removes lesions anywhere in DNA and is initiated by binding of the XPC-RAD23B-CETN2 complex to damaged DNA (20). Both damage detection mechanisms converge on a common core NER pathway that verifies the presence of lesions and excises the DNA damage followed by gap-filling DNA synthesizes and ligation (14). Notably, XPC does not directly bind to DNA damage to initiate GG-NER but recognizes lesion-induced helical distortions by interacting with the non-damaged DNA strand and inserting a β-hairpin domain into the DNA duplex (21,22). Because of this, XPC can detect a broad diversity of DNA lesions, although certain lesions are detected more efficiently than others (23). XPC efficiently binds to UV-induced 6–4 pyrimidine–pyrimidone photoproducts (6–4PPs) but only weakly recognizes UV-induced cyclobutane-pyrimidine dimers (CPDs), since these cause only minor DNA helix distortion. For these difficult-to-detect lesions, XPC functions together with the E3 ubiquitin ligase complex CRL4^DDB2^, consisting of CUL4A, RBX1, DDB1 and DDB2 (24–26). DDB2 directly binds to, and flips out, damaged bases to create a more suitable DNA substrate for XPC (27–29). Also, DDB2 is thought to be important in orchestrating chromatin reorganization during GG-NER (30). Damage detection by DDB2 and XPC is tightly regulated by post-translational modifications such as ubiquitylation, as CRL4^DB2^ ubiquitylates both proteins to regulate DNA damage binding and handover from DDB2 to XPC by controlling their DNA association and dissociation (29,31–34). Several studies showing the affinity of DDB2 to lesions such as apurinic sites, mismatches and oxidative lesions suggest that the specificity of DDB2 is broader than only the recognition of UV photolesions (35,36). It is, however, unknown whether the function of DDB2 is also relevant for detection of other NER substrates, such as oxaliplatin- and cisplatin-DNA lesions. Moreover, how the assessment of patient NER activity could be used clinically, to predict and improve cancer treatment by platinum-based chemotherapy, remains largely uninvestigated (37).

Our previous CRISPR/Cas9-based genetic screen demonstrated the relative importance of TC-NER in the repair of oxaliplatin and cisplatin-induced DNA damage in DLD1-colon cancer cells (16). However, against expectation, this screen suggested that GG-NER is not required for the clearance of oxaliplatin-induced DNA lesions. Here, we show that some GG-NER proficient colon cancer cells completely lack the ability to clear oxaliplatin-induced DNA lesions. Using a combination of live cell imaging and genetic screening, we show that XPC alone inefficiently binds to oxaliplatin lesions because it requires DDB2 and HMGA2 to effectively initiate GG-NER. Hence, we show that GG-NER of oxaliplatin-induced DNA lesions requires auxiliary factors and is therefore more variable between cancer cells in comparison to the clearance of cisplatin-induced DNA lesions. Finally, we propose that this variability can be utilized in cancer treatment strategies and we identify DDB2 as a new promising predictive marker of oxaliplatin efficiency in colorectal cancer.

## Materials and methods

### Cell culture

All cells were maintained at 37°C, in 20% O_2_ and 5% CO_2_ and regularly checked for mycoplasma. Colon cancer cells DLD-1 and HCT116 were purchased from Horizon Discovery. HT-29, HCT-15, COLO 205, LoVo, Caco-2 and SW620 were a kind gift from Riccardo Fodde (Erasmus MC Rotterdam, The Netherlands) and RKO cells were a kind gift from Georg Winter, CeMM Vienna, Austria). Cells were cultured in a 1:1 mixture of Roswell Park Memorial Institute-1640 medium (RPMI, Sigma-Aldrich) and Ham's F-10 nutrient mix (Lonza), supplemented with 10% fetal bovine serum (FBS; Gibco), and 1% penicillin/streptomycin (PS; Sigma-Aldrich). Flag-GFP-XPC knock-in (KI) HCT116 cells (hereafter referred to as GFP-XPC HCT116), a kind gift from Jurgen Marteijn (Erasmus MC Rotterdam, The Netherlands; (38), were cultured in RPMI media supplemented with 20% AmnioMAX™-II Complete Media (Thermo Fisher Scientific), 10% FBS and 1% PS. HEK293T cells were obtained from the Cancer Research UK London Research Institute Cell Facility and were used for virus production, by culturing in Dulbecco's modified Eagle media (Sigma-Aldrich) and supplemented with 10% FBS and 1% PS.

### Chemicals

For all experiments, except those shown in Supplementary Figure S6, oxaliplatin and cisplatin (Sigma-Aldrich) were dissolved in phosphate-buffered saline (PBS) to 10 mM, aliquoted and stored at -20°C until use. For experiments shown in Supplementary Figure S6c and d, oxaliplatin and cisplatin were freshly dissolved in 0.9% NaCl and in Supplementary Figure S6e, oxaliplatin was freshly dissolved in 5% dextrose (Sigma) solution. Chemical concentrations and treatment durations are indicated for each experiment. Deubiquitinase inhibitor Pierce™ NEM (*N*-ethylmaleimide; Thermo Fisher) was freshly prepared before each experiment by resuspending in absolute ethanol.

### Generation of knock-out cells

To generate stable XPC knock-out (KO) colon cancer cells, plentiCRISPRv2 (gift from Feng Zhang; Addgene plasmid # 52961 (39), carrying sgRNAs targeting XPC (Supplementary Table S1) together with Lipofectamine™ 2000 Transfection Reagent (Thermo Fisher Scientific) was transiently transfected into cells according to the manufacturer's protocol. Twenty-four hours after transfection, cells were selected with puromycin and clones were isolated. Loss of XPC expression was analysed by immunoblotting. To generate HMGA2 and DDB2 depletion in HCT116 GFP-XPC cells, 2 sgRNAs targeting different exons of each respective gene were designed using the VBC score (40) (Supplementary Table S2) and were *in-vitro* transcribed (IVT) as previously described (41). Both IVT sgRNAs targeting the same gene were then nucleofected together with purified Cas9 protein (Integrated DNA Technologies) into 0.2 M cells in 16-well strips, using a 4D Nucleofector (Lonza) according to the manufacturer's protocol. IVT-nucleofected cell populations were then clonally expanded and sgRNA-target sequences were analyzed for the presence of insertions/deletions (indels). To do so, genomic DNA was isolated using DNeasy Blood & Tissue Kit (Qiagen) and fragments spanning the sgRNA target sites were amplified by PCR using GoTaq DNA Polymerase (Promega) and primers listed in Supplementary Table S2. For PCR purification, 5 μl of PCR product were incubated with 1 unit of rSAP phosphatase and 20 units of EXO1 exonuclease (New England Biolabs) for 5 min at 37°C followed by 10 min at 80°C. PCR products were then Sanger sequenced (Microsynth), after which indel frequency was calculated using the Tracking of Indels by DEcomposition (TIDE) algorithm (42).

### Generation of transient overexpression and knock-in cells

To generate DLD-1 and HCT116 XPC KO cells transiently expressing XPC-GFP, cells were transfected with an XPC-GFP cDNA construct (pLenti-CMV-Puro-DEST; (43)) using jetPEI® transfection reagent (Polyplus) following the producer's protocol. To generate GFP-DDB2 HCT116 KI cells, cells were transfected with pLentiCRISPR-v2 carrying an sgRNA targeting the start codon of the DDB2 locus (target sequence CCTTCACACGGAGGACGCGA), and a DNA construct of GFP flanked by 60 bp sequences homologous to the DDB2 locus. After selection with puromycin and FACS for GFP-positive cells, a clonal cell line was isolated and verified by sequencing and functional analysis.

### Arrayed CRISPR screen

The performance of the initial CRISPR genetic screen for the identification of DDR genes that confer hypersensitivity of DLD1 cells to oxaliplatin is shown in Supplementary Figure S1a and described in detail (16). The secondary screen was performed in HCT116 cells and was designed to confirm the genes involved in oxaliplatin hypersensitivity based on their loss of expression in XPC^OXA^-inactive cells (identified through RNA sequencing – see below). Here, 35 candidate genes were targeted by 3 different sgRNA’s, the sequences of which were either taken from Toronto KnockOut CRISPR Library Version 3, where they were reported to have a guide score > 1.0 (Addgene #90294; (44)) or designed by the CHOPCHOP web tool (45) (Supplementary Table S3). *DDB2* was included as a positive control and two sgRNAs complementary to non-coding regions were used as negative controls. sgRNAs were individually cloned into the plentiCRISPRv2 vector and transformed into competent bacteria. Bacteria cultures with amplified sgRNAs targeting the same gene were then pooled and plasmids were isolated by QIAprep Spin Miniprep Kit (Qiagen). 1 μg vector DNA was transfected into HEK 293T cells growing in 6-well plate format using Lipofectamine™ 2000 Transfection Reagent (Thermo Fisher Scientific), together with 0.5 μg VSV-G, and 0.5 μg psPAX2, to generate lentiviruses. Lentiviral particles were harvested 48 h after transfection and used to transduce HCT116 cells in a 12-well plate format for 24 h. Transduced cells were then selected with puromycin and immediately used for survival experiments or cryopreserved.

### RNA sequencing and data processing

DLD-1, LoVo, HCT116 and SW480 cells were grown in 6-well plates before total RNA isolation was performed using the RNeasy kit (Qiagen) in biological triplicates. The amount of total RNA was quantified using the Qubit 2.0 Fluorometric Quantitation system (Thermo Fisher Scientific) and the RNA integrity number (RIN) was determined using the Experion Automated Electrophoresis System (Bio-Rad). RNA-seq libraries were prepared with the TruSeq Stranded mRNA LT sample preparation kit (Illumina) using Sciclone and Zephyr liquid handling workstations (PerkinElmer) for pre- and post-PCR steps, respectively. Library concentrations were quantified with the Qubit 2.0 Fluorometric Quantitation system (Life Technologies) and the size distribution was assessed using the Experion Automated Electrophoresis System (Bio-Rad). For sequencing, samples were diluted and pooled into NGS libraries in equimolar amounts. Expression profiling libraries were sequenced on HiSeq 3000/4000 instruments (Illumina) following a 50-bp, single-end recipe. Raw data acquisition (HiSeq Control Software, HCS, HD 3.4.0.38) and base calling (Real-Time Analysis Software, RTA, 2.7.7) was performed on-instrument, while the subsequent raw data processing off the instruments involved two custom programs (46) based on Picard tools (2.19.2) (47). In a first step, base calls were converted into lane-specific, multiplexed, unaligned BAM files suitable for long-term archival (IlluminaBasecallsToMultiplexSam, 2.19.2-CeMM). In a second step, archive BAM files were demultiplexed into sample-specific, unaligned BAM files (IlluminaSamDemux, 2.19.2-CeMM). NGS reads were mapped to the Genome Reference Consortium GRCh38 assembly via ‘Spliced Transcripts Alignment to a Reference’ (STAR) (48) utilising the ‘basic’ Ensembl transcript annotation from version e100 (April 2020) as reference transcriptome. Since the hg38 assembly flavor of the UCSC Genome Browser was preferred for downstream data processing with Bioconductor packages for entirely technical reasons, Ensembl transcript annotation had to be adjusted to UCSC Genome Browser sequence region names. STAR was run with options recommended by the ENCODE project. Aligned NGS reads overlapping Ensembl transcript features were counted with the Bioconductor (3.12) GenomicAlignments (1.26.0) package via the summarizeOverlaps function in Union mode, considering that the Illumina TruSeq stranded mRNA protocol leads to sequencing of the first strand so that all reads needed inverting before counting. Transcript-level counts were aggregated to gene-level counts and the Bioconductor DESeq2 (1.30.0) (49) package was used to test for differential expression based on a model using the negative binomial distribution. An initial exploratory analysis included principal component analysis (PCA), multi-dimensional scaling (MDS), sample distance and expression heatmap plots, all annotated with variables used in the expression modelling (ggplot2, 3.3.3; (50) and Bioconductor Complex Heatmap, 2.6.2; (51,52)), as well as volcano plots (Bioconductor Enhanced Volcano, 1.8.0; (53)). Biologically meaningful results were extracted from the model, log2-fold values were shrunk with the CRAN ashr (2.2.-47) (54) package, while two-tailed *P*-values obtained from Wald testing were adjusted with the Bioconductor Independent Hypothesis Weighting (IHW, 1.18.0) package (55). The resulting gene lists were annotated, filtered for significantly differentially up- and down-regulated genes and independently subjected to gene set enrichment analysis (Enrichr) (56).

### Cell viability and survival assays

All cellular survival responses to oxaliplatin, cisplatin or ultraviolet light (UV) were measured by MTT CellTiter 96® non-radioactive cell proliferation assay or CellTiter-Glo 2.0 Assay (Promega), except for the clonogenic survival experiments shown in Supplementary Figure S6c and d. For MTT assays, cells were seeded at a density of 2000 cells per well in 96-well plates in triplicates in media supplemented with the respective concentrations of drugs, as indicated in each experiment. Cells were then allowed to grow for 3 days before cellular viability evaluation. In the case of UV, cells were grown in 6-well plates, washed with PBS and irradiated with the respective dose of 254 nm UV-C using a UVP crosslinker CL-3000 (Analytik Jena). After that, cells were trypsinized and seeded at a density of 2000 cells per well in 96-well plates in triplicates in normal media. Residual cell viability was measured by adding MTT or CellTiter-Glo solution to the media following the manufacturer's recommendations. Absorbance at 490 nm was measured using GloMax®-Multi Detection System (Promega). Luminescence was evaluated using the plate reader Infinite 200 PRO (Tecan Life Sciences). Clonogenic survival assays were performed by seeding 500 cells per 6 cm dish in duplicate. The next day, cells were treated with freshly prepared oxaliplatin or cisplatin solution in 0.9% NaCl at the indicated doses for 2 h. The medium was refreshed and after 7 days, cells were fixed and stained with methylene blue, after which colonies were counted. Raw data were analyzed by Prism software version 9.2.0 (GraphPad). Relative cell viabilities were calculated as a percentage of untreated control of each cell line and, for MTT assays, plotted against log(10) transformed drug concentrations. Lethal doses of 50% (LD50) were calculated by nonlinear regression curve fit.

### Cellular platinum uptake

DLD-1 and HCT116 cells were grown in 6-well plates and treated with 10 μM or 50 μM oxaliplatin or cisplatin for 6 h in technical triplicates. Empty wells with no cells but treated with drugs were prepared in parallel as a blank control for measuring the background signal. After the treatment, cells were thoroughly washed with PBS 3 times, lysed in 500 μl of nitric acid (≥69%, p.a., HPLC grade, Fluka) and incubated for 1 h at room temperature. 400 μl of lysate from each well was diluted 20-fold in dd H_2_O. Platinum content was determined by inductively coupled plasma mass spectrometry (ICP-MS) using an ICP-MS Agilent 7800® (Agilent Technologies) equipped with an Agilent SPS 4 autosampler (Agilent Technologies) and a MicroMist nebulizer at a sample uptake rate of approx. 0.2 ml/min. Ultrapure water (18.2 MΩ cm, Milli-Q Advantage) was used for all dilutions for ICP-MS measurements. Platinum and rhenium standards were derived from CPI International. The Agilent MassHunter® software package (Workstation Software, version C.01.04, Build 544.17, Patch 3, 2018) was used for data processing. The experimental parameters for ICP-MS are specified in Supplementary Table S4. The instrument was tuned daily to achieve maximum sensitivity. Final platinum content was expressed as ng platinum per 10^6^ cells by subtracting the blank controls and normalizing the measured amount of platinum to the number of cells counted in the 6-wells for a respective cell line.

### Dot blot

After treatment with 100, 200, 400, 600 and 800 μM of oxaliplatin or cisplatin for 6 h, DLD-1 and HCT116 cells were harvested with trypsin, and DNA was extracted using the QIAamp DNA Blood Mini Kit (Qiagen) using the manufacturer's instructions. After evaluating the DNA concentration by Qubit 2.0 Fluorometric Quantitation system (Thermo Fisher Scientific), DNA was denatured by addition of 0.4 M NaOH and 10 mM EDTA and incubated at 100°C for 10 min. This was followed by neutralization in cold 2 M ammonium acetate at pH 7.0. Blotting was performed using a nitrocellulose membrane using a Bio-Dot apparatus (Bio-rad). After baking of the membrane for 2 h at 80°C under a vacuum, the membrane was washed once with TBS-T and blocked with 5% milk in TBS-T for 1 h at room temperature. The membrane was incubated with anti-cisplatin modified DNA antibody [CP9/19] (1:1000, Abcam, ab103261) overnight at 4°C. The membrane was washed 3x with TBS-T for 10 min and incubated with anti-rat-HRP antibody (1:5000, Jackson ImmunoResearch, 112-035-003) for 1 h at room temperature, followed by 3x washes with TBS-T 10 min each wash. Visualization was achieved using the Amersham ECL Western Blotting Detection Reagent (Sigma-Aldrich) and developed using a Curix 60 (AGFA) table-top processor. Evaluation of DNA loading was performed using SYBR Gold Nucleic Acid Gel Stain (Thermo Fisher Scientific) and imaged using the ChemiDoc Imaging System (Bio-rad). Quantification of crosslinks was performed by ImageJ software (free version, NIH) by normalizing total antibody intensities to the respective intensities of SYBR Gold for each data point.

### Immunoprecipitation

HCT116 GFP-XPC cells were grown in 15 cm plates and treated with 600 μM of oxaliplatin or cisplatin for 2, 4 or 6 h or irradiated with 30 J/m^2^ UV-C and further cultured for 30 min to allow for DNA repair. Cells were then washed with PBS and crosslinked for 10 min with 1% formaldehyde in serum-free media at room temperature while shaking. The reaction was terminated by adding 125 mM glycine for 5 min. After 3-times washes with ice-cold PBS, cells were scraped and spun at maximum speed for 15 min at 4°C in a desk-top centrifuge. Pellets were resuspended in 1 ml/plate Buffer 1 (50 mM HEPES pH 7.8, 150 mM NaCl, 1 mM EDTA, 0.5 mM EGTA, 0.25% Triton-X, 0.5% NP-40, 10% Glycerol, 1 mM PMSF, 2.5× Thermo Scientific™ Halt™ Protease Inhibitor Cocktail (Thermo Fisher Scientific) and incubated for 30 min with constant rotation at 4°C. Samples were spun at 2000 rpm, 5 min at 4°C. The nuclear pellets were then resuspended in 5x volume of Buffer 2 (10 mM Tris pH 8.0, 200 mM NaCl, 1 mM EDTA, 0.5 mM EGTA, 1mM PMSF, 2.5x Thermo Scientific™ Halt™ Protease Inhibitor Cocktail (Thermo Fisher Scientific) and rotated 15 min at 4°C, followed by centrifugation at 2000 rpm at 4°C. After a second wash with Buffer 2, pellets were then resuspended in 5× volume of RIPA buffer (25 mM Tris pH 8.0, 150 mM NaCl, 0.1% SDS, 1% NP-40, 0.5% sodium deoxycholate, 5 mM EDTA, 1mM PMSF, 2.5x Thermo Scientific™ Halt™ Protease Inhibitor Cocktail (Thermo Fisher Scientific). Samples were thoroughly sonicated by 12 cycles of 10 s on 10 s off with a power of 25% and centrifuged at maximum speed in a desk-top centrifuge for 15 min at 4°C. The protein concentration of the supernatants was evaluated using Protein Assay Dye Reagent (Biorad) and an equal concentration of proteins were incubated with GFP-Trap® beads (Chromotek) overnight at 4°C. After that, samples were centrifuged at 2000 rpm at 4°C and thoroughly washed 5-times with RIPA buffer. Finally, pellets were resuspended in 2% SDS HEPES buffer (50 mM HEPES pH 8.0, 2% SDS, 100 mM DTT, 2× Thermo Scientific™ Halt™ Protease Inhibitor Cocktail and incubated at 37°C for 30 min for elution of proteins. After a 5 min spin at 2000 rpm, supernatants were transferred to a new vial and boiled at 95°C for 25 min to revert protein crosslink, before analyzing the samples by immunoblotting.

### Immunoblotting

Cells were washed with PBS and lysed in RIPA lysis buffer (New England Biolabs) containing 1 mM PMSF and EDTA-free protease inhibitor cocktail (Roche). To visualize XPC-ubiquitylation, 20 mM of NEM deubiquitinase inhibitor was added to the RIPA buffer. Lysates were sonicated and protein concentrations were measured using the Protein Assay Dye Reagent (Biorad). Samples were then mixed with NuPAGE LDS Sample Buffer (Invitrogen) and boiled for 5 min at 98°C. Proteins were separated on SDS-PAGE gels and transferred onto Amersham™ Protran nitrocellulose membranes (0.45μm, Cytiva) or onto Amersham™Hybond PVDF membranes (0.2μm, Cytiva). After 1 h of blocking in 5% milk in TBS-T (0.1% Tween 20 in Tris-buffered saline), membranes were incubated with primary antibodies at 4°C overnight. Primary antibodies used were anti-XPC D-10 (1:1000, Santa Cruz, SACSC-74410), anti-XPC (1:2000, Bethyl Laboratories, A301-121A), anti-DDB2 [EPR9811] (1:1000, Abcam, ab181136), anti-UBA2 B-6 (1:1000, Santa Cruz, sc-376305), anti-HMGA2 D1A7 (1:300, Cell Signaling, 8179S), anti-MSH6 44/MSH6 (RUO) (1:1000, BD Bioscience, 610918). As loading controls anti-Ku70 M-19 (1:1000, Santa Cruz, sc-1487) and anti-Tubulin DM1A (1:10000, Cell Signaling, mAb #3873) were used. Anti-mouse and anti-rabbit HRP-conjugated goat secondary antibodies (Jackson Immunochemicals) were used at a final dilution of 1:5000. Immunoblots were incubated with Amersham ECL Western Blotting Detection Reagent (Sigma-Aldrich) and imaged with Curix 60 (AGFA) table-top processor. Relative protein concentrations normalized to a loading control were evaluated by ImageJ (free version, NIH).

### Fluorescence recovery after photobleaching

Fluorescence recovery after photobleaching (FRAP) was used to measure the mobility of plenti-XPC-GFP transiently expressed in DLD-1 or HCT116 XPC KO cells, GFP-DDB2 KI in HCT116 cells and GFP-XPC KI in HCT116 cells, using an SP5 or SP8 confocal laser-scanning microscope (Leica). For experiments shown in Supplementary Figure S6e, cells were transfected with control siRNA (UGGUUUACAUGUUGUGUGA, Dharmacon) or siRNA targeting HMGA2 (CCUAAGAGACCCAGGGGAA, Dharmacon) 48 h before the FRAP experiment, using Lipofectamine RNAiMax (Invitrogen) according to the manufacturer's protocol. Cells were untreated or treated for 6 h with 400 or 600 μM of oxaliplatin or 200–600 μM of cisplatin or 10 or 15 J/m^2^ of UV-C, as indicated for each experiment. Immediately after the treatment, GFP fluorescence was monitored every 22 ms at 1400 Hz with a magnification of 12× in the strip of 512 × 16 pixels, until a steady state level was reached. Next, a strip was photobleached for 176 ms at maximum laser intensity and recovery of the fluorescence was recorded using low laser intensity every 22 ms for a total of 35 s, until steady-state levels were reached. FRAP data were background-corrected and normalized to the average pre-bleach fluorescence levels set at 100%. At least two independent experiments of > 12 cells were performed for each condition. The immobile fraction (*F*_imm_) for each treatment condition was determined by normalizing the measurements to the fluorescence intensity recorded immediately after bleaching (*I*_0_) and the average fluorescence of the last 200 frames of the post-bleach phase (once recovery reached steady-state) from the untreated cells (*I*_final, untr_) and treated cells (*I*_final, treat_), using the formula: *F*_imm_ = 1 × (*I*_final, treat ×_ *I*_0, treat_)/(*I*_final, untr ×_ *I*_0, treat_) (33).

### Patient survival data

Survival analyses were performed using the R software for statistical computing (57). Clinical data from cancer patients as well as the normalized gene expression levels (fragments per kilobase of exon per million fragments mapped, FPKM) were downloaded from The Cancer Genome Atlas (TCGA). Next, FPKM values were log2-transformed and mean-centered. The expression of each gene was tested along with survival data with logrank tests and the Cox proportional hazards regression model, both available in the R package survival (58,59). In the first case, an algorithm was used to search for the cutoff in expression leading to the separation of groups with more contrast in overall survival (function maxstat.test from the package maxstat; (60). A *P*-value cutoff of 0.05 was adopted to select genes whose expression levels were significantly related to survival.

### Statistical analysis

Mean values and S.E.M. error bars are shown for each experiment. In the cell survival data, concentrations of drugs were log10 transformed and data were normalized to untreated controls. LD50 concentrations of drugs were calculated from nonlinear fit log(inhibitor) versus normalized response variable slope. Differences were calculated using a two-tailed *t*-test. Unpaired two-tailed parametric *t*-test with Welch's correction without assuming a consistent standard deviation was applied for comparison of groups in FRAP analysis. All analyses were performed using Prism 9 for macOS software (GraphPad).

## Results

### A subset of colon cancer cells is deficient in the removal of oxaliplatin-DNA lesions by GG-NER

**Figure 1.**
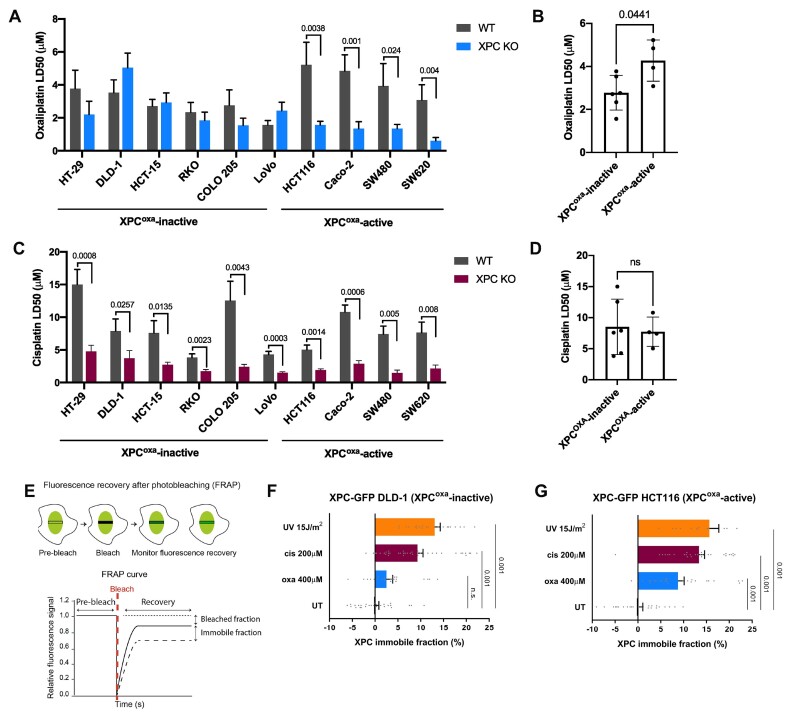
A subset of colon cancer cells is deficient in the removal of oxaliplatin-DNA lesions by GG-NER. (**A**) Sensitivity of ten XPC KO colon cancer cell lines and their WT counterparts to oxaliplatin, expressed as drug lethal dose required for 50% cell death of the cell population (LD50). Cell lines with no difference between XPC KO and WT are grouped as XPC^oxa^-inactive, while cell lines where XPC loss significantly elevated sensitivity to oxaliplatin are grouped as XPC^oxa^-active. (**B**) LD50 oxaliplatin values of XPC^oxa^-inactive and XPC^oxa^-active WT colon cancer cell lines. (**C**) Cisplatin LD50 values of XPC KO and WT colon cancer cell lines. (**D**) Cisplatin LD50 values of XPC^oxa^-inactive and XPC^oxa^-active WT colon cancer cell lines. (**E**) Scheme depicting the fluorescence recovery after photobleaching (FRAP) assay to measure protein binding to chromatin. Fluorescent signal of a fluorescently-tagged protein is bleached with high laser power in a small strip spanning the cell nucleus, subsequent fluorescence recovery is determined over time as a measure for protein mobility. Incomplete recovery after DNA damage induction indicates that a percentage (‘the immobile fraction’) of protein molecules is bound to DNA and active in DNA repair. (**F**) Immobile fraction of XPC represents its binding to chromatin after induction of DNA damage. XPC immobilization was determined by FRAP of XPC-GFP transiently expressed in XPC-deficient DLD-1 cells (representative of an XPC^oxa^-inactive cell line) and (**G**) HCT116 cells (representative of an XPC^oxa^-active cell line). Cells were untreated (UT) or treated for 6 h with 400 μM of oxaliplatin (oxa), 200 μM of cisplatin (cis*)*or irradiated with 15 J/m^2^ UV. Data in A and C represent mean +/− SEM of three independent experiments. Data in B and D represent mean +/− SD of WT cells from A and B, respectively. LD 50 concentrations for each cell line and each drug were calculated from dose-response curves presented in Supplementary Figure S2a and b. Differences were calculated using the two-tailed t-test. Each dot in the FRAP graphs represents a single cell. A minimum of 15 cells were measured per experimental condition. Unpaired two-tailed parametric t-test with Welch's correction without assuming a consistent standard deviation was applied for comparison of groups in FRAP analysis.

To characterize the DDR to oxaliplatin, we previously performed a loss-of-function CRISPR/Cas9 screen to identify major DNA repair factors that are required for survival of colon cancer DLD-1 cells exposed to oxaliplatin. The screen showed that genes of the FA, TLS, HR, BER and TC-NER pathways are important to protect cells against oxaliplatin-induced DNA lesions, which is more elaborately described in our previous study (16). However, we observed that DLD-1 cells expressing sgRNAs targeting the core GG-NER factor XPC did not show elevated sensitivity to oxaliplatin as compared to their wild-type (WT) counterparts (Supplementary Figure S1a and (16)). This indicates that DLD-1 cells do not utilize XPC for oxaliplatin-DNA lesions repair, which is unexpected, since XPC is the main DNA damage recognition factor for bulky lesions including platinum-DNA intrastrand crosslinks (14,15,61). To validate this result, we abrogated XPC function by CRISPR/Cas9 in nine other colon cancer cell lines, namely HT-29, HCT-15, RKO, COLO205, LoVo, HCT116, Caco2, SW480 and SW620. Loss of XPC protein was verified by immunostaining (Supplementary Figure S1b) and by showing hypersensitivity to UV irradiation (Supplementary Figure S1c). We treated the panel of colon cancer cells and their isogenic XPC knock-out (KO) counterparts with oxaliplatin and observed that in 6 out of 10 cell lines XPC loss did not lead to increased sensitivity to oxaliplatin (Figure 1A and Supplementary Figure S2a). Moreover, these 6 cell lines (HT-29, DLD-1, HCT-15, RKO, COLO205 and LoVo, hereafter ‘XPC^oxa^-inactive’ cell lines) were on average more sensitive to oxaliplatin, as represented by their lower LD50 values, in comparison to cell lines which became hypersensitive to oxaliplatin in the absence of XPC (HCT116, Caco2, SW480 and SW620, hereafter ‘XPC^oxa^-active’) (Figure 1B). These results suggest that XPC, and thus GG-NER, is not active in the removal of oxaliplatin-DNA lesions in a subset of colon cancer cells and that oxaliplatin exhibits elevated cytotoxicity when GG-NER is not engaged. To investigate whether these cell lines similarly do not engage GG-NER after exposure to other platinum crosslinkers, cells were treated with cisplatin. In contrast to oxaliplatin, cisplatin triggered elevated lethality in all studied XPC KO cell lines when compared to their WT cognates (Figure 1C and Supplementary Figure S2b). Also, LD50 concentrations of cisplatin did not differ between XPC^oxa^-inactive and -active groups (Figure 1D). These data indicate that the inability to recognize DNA damage by GG-NER is specific to DNA lesions generated by oxaliplatin.

DNA damage recognition and initiation of GG-NER by XPC is reflected by its binding to chromatin after DNA damage induction, which can be quantitatively determined in living cells by measuring its mobility by fluorescence recovery after photobleaching (FRAP) (Figure 1E; (33,62)). We used DLD-1 cells as representative for the XPC^oxa^-inactive cell lines, and HCT116 cells for the XPC^oxa^-active cell lines. DLD-1 and HCT116 cells depleted of endogenous XPC were transiently transfected with XPC-GFP cDNA and XPC binding to different types of lesions induced by UV, cisplatin and oxaliplatin was measured. In FRAP with oxaliplatin and cisplatin, in contrast to UV irradiation (33,62), high, non-physiological doses need to be used to induce sufficient DNA damage levels to be able to detect the binding of XPC molecules to platinum-DNA lesions, as previously also observed for DNA repair proteins CSB and XRCC1 (16). While in HCT116 cells XPC was clearly immobilized following each type of treatment, in DLD-1 cells XPC was only immobilized after UV and cisplatin but not after oxaliplatin treatment (Figure 1F and G). These data are in line with the dose-response data (Figure 1A and C) and demonstrate that the lack of XPC activity in response to oxaliplatin is not due to impaired XPC activity in these cells.

To investigate if uptake of oxaliplatin is impaired in XPC^oxa^-inactive cell lines, we treated DLD-1 and HCT116 cells with cisplatin or oxaliplatin and quantified cellular platinum uptake by ICP-MS and DNA-crosslink generation by dot blot analysis. For dot blot analysis, we used an antibody raised against cisplatin-DNA lesions, which predominantly recognizes intrastrand crosslinks between adjacent guanines, but not ICLs (63), and which was also shown to recognize similar lesions induced by oxaliplatin (64). These intrastrand crosslinks are the predominant type of lesions induced by cisplatin and oxaliplatin and are specifically detected and repaired by NER (15,61,65). As other intrastrand crosslinks and ICLs are formed in the same relative proportion to these crosslinks, the signal from this antibody correlates to overall DNA damage levels. We observed that in XPC^oxa^-inactive DLD-1 cells, oxaliplatin uptake and its ability to generate DNA intrastrand crosslinks did not differ from XPC^oxa^-active HCT116 cells (Supplementary Figure S3a–c). We therefore excluded this as an explanation for why these cells have different XPC activities.

Taken together, we showed that oxaliplatin-DNA crosslinks are not recognized by XPC and therefore not repaired by GG-NER in a subpopulation of colon cancer cells. We hypothesize that this is likely due to the different genetic backgrounds of the cell lines.

### DDB2 promotes efficient XPC-mediated recognition of oxaliplatin-DNA lesions

We reviewed known genetic characteristics of the panel of 10 colon cancer cells (66) to identify possible genetic alterations that could explain the differential responses of XPC^oxa^-active and XPC^oxa^-inactive cell lines. Four of the XPC^oxa^-inactive cell lines have microsatellite instability caused by deficiency in mismatch repair, and mismatch repair has been implicated in the response to DNA crosslinks (67,68). However, also the XPC^oxa^-active HCT116 cell line is mismatch repair deficient. Therefore, we chose not to focus on mismatch repair. Also, none of the other commonly evaluated oncogenic features, such as, chromosomal instability, CpG island methylation or mutations in genes *TP53, KRAS, BRAF, PIK3CA* or *PTEN* clearly correlated with the cellular response to oxaliplatin (Supplementary Figure S4a).

**Figure 2.**
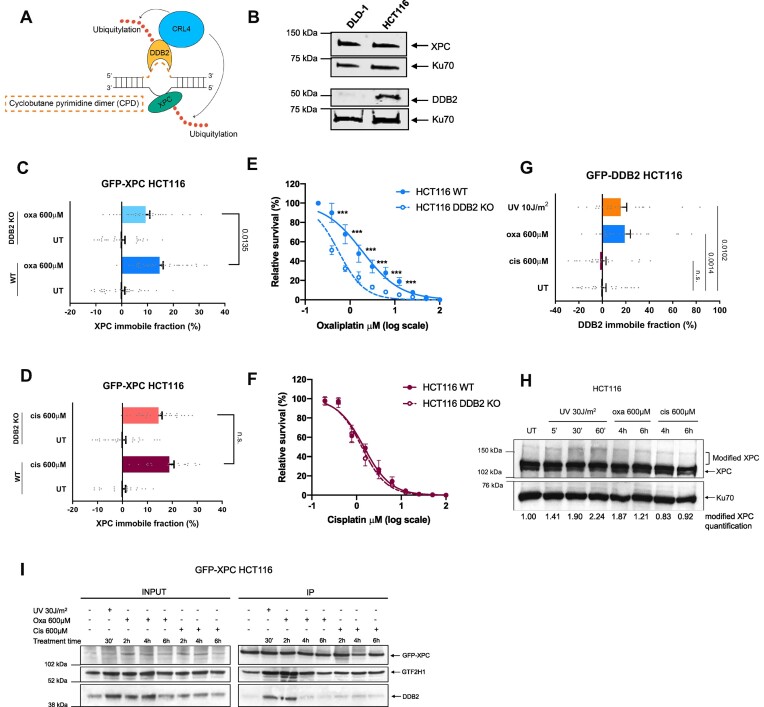
DDB2 promotes efficient XPC-mediated recognition of oxaliplatin-DNA lesions. (**A**) Scheme depicting the recognition of UV-induced cyclobutane pyrimidine dimers (CPDs) by GG-NER. The DDB2 protein, which is bound to the CRL4 E3 ubiquitin ligase complex consisting of CUL4A, DDB1 ad RBX1, binds to CPDs and increases XPC affinity for DNA lesions by ubiquitylation. Upon XPC recruitment and binding of lesions, GG-NER is initiated. (**B**) Immunoblot showing XPC and DDB2 total protein levels in protein lysates of untreated DLD-1 and HCT116 cells. Ku70 was stained as a loading control. (**C**) FRAP analysis showing XPC chromatin binding in WT and DDB2 KO GFP-XPC KI HCT116 cells untreated (UT) or treated for 6 h with 600 μM of oxaliplatin or (**D**) 600 μM of cisplatin. (**E**) Percentage survival of HCT116 WT and DDB2 KO cells to increasing doses of oxaliplatin or (**F**) cisplatin. (**G**) FRAP analysis showing DDB2 chromatin binding in DDB2 KO GFP-DDB2 KI HCT116 cells untreated (UT) or treated for 6 h with 600 μM oxaliplatin or cisplatin or with 10 J/m^2^ UV. (**H**) Immunoblot showing XPC ubiquitylation in HCT116 cells untreated (UT), treated for 4 or 6 h with 600 μM of oxaliplatin or cisplatin, or, as control, irradiated with 30 J/m^2^ UV and harvested at the indicated time points. Ubiquitylated forms of XPC are labeled as ‘modified XPC’, Ku70 was used as a loading control. Numbers under the immunoblot indicate quantified total pixel intensity of the ‘modified XPC’ bands normalized to loading control and the untreated condition. (**I**) Immunoblot of XPC immunoprecipitation (IP) in GFP-XPC KI HCT116 cells treated with 600 μM of oxaliplatin or cisplatin for the indicated times or with 30 J/m^2^ UV followed by a period of repair of 30 min. Each dot in the FRAP graphs represents a single cell. A minimum of 20 cells were measured per experimental condition. Unpaired two-tailed parametric t-test with Welch's correction without assuming a consistent standard deviation was applied for comparison of groups in the FRAP analysis. Data in (E) and (F) represent mean +/− SEM of 3 independent experiments. Differences were calculated using the two-tailed *t*-test. *** *P*-value < 0.001.

To further investigate the absence of oxaliplatin-DNA lesion recognition by XPC in a subset of colon cancer cell lines, we focused on the GG-NER pathway. The CRL4^DDB2^ complex facilitates the detection of UV-induced CPDs by XPC by binding to, and flipping out, damaged bases and by ubiquitylation of XPC (Figure 2A), but it is unknown whether it has a similar role in the detection of platinum-DNA lesions. Therefore, we compared the total XPC and DDB2 protein levels in DLD-1 XPC^oxa^-inactive cells and HCT116 XPC^oxa^-active cells. We observed reduced DDB2 levels in DLD-1 as compared to HCT116 cells (Figure 2B). Lower DDB2 levels were found in most other XPC^oxa^-inactive colon cancer cell lines (Supplementary Figure S4b–d). This may indicate that in some of these XPC^oxa^-inactive cells, low DDB2 levels are a limiting factor for efficient recognition of oxaliplatin-DNA lesions. To address this, we used previously generated GFP-XPC knock-in HCT116 cells and verified GFP-XPC activity after UV and platinum drugs treatment by FRAP (Supplementary Figure S4e). Next, we knocked out DDB2 to measure XPC chromatin binding by FRAP after treatment with platinum drugs in the presence and absence of DDB2. Loss of DDB2 had no significant effect on XPC mobility in cisplatin-treated cells, but it reduced the immobile XPC fraction in cells treated with oxaliplatin (Figure 2C and D). This suggests that XPC partly depends on DDB2 for efficient recognition and repair of a fraction of oxaliplatin-DNA lesions. We then tested the sensitivity of DDB2 KO cells to both platinum drugs. In agreement with the FRAP data, DDB2-depleted cells showed elevated sensitivity to oxaliplatin but not to cisplatin, compared to WT cells (Figure 2E and F). Finally, we generated GFP-DDB2 knock-in HCT116 cells and directly tested DDB2 immobilization in response to both types of platinum lesions by FRAP, compared to UV-irradiation that served as a positive control. In agreement with the above data, DDB2 was immobilized in response to oxaliplatin and UV, but not to cisplatin (Figure 2G).

A hallmark of DDB2 involvement in GG-NER of UV-induced DNA lesions is the DNA damage-induced ubiquitylation of XPC by the CRL4^DDB2^ E3 ubiquitin complex (25,32). We therefore determined if XPC is ubiquitylated after oxaliplatin but not after cisplatin exposure. XPC ubiquitylation, and other XPC modifications like SUMOylation, can be visualized by western blot and appear as higher molecular weight bands above XPC (69). The appearance of these bands after UV irradiation depends on DDB2 (33), and their intensity changes upon inhibition of the proteasome or inhibition or depletion of the ubiquitin-dependent segregase VCP/p97 or the deubiquitylase USP7 (34,70). In line with these previous findings, we clearly detected modified XPC bands 30 and 60 min after UV-irradiation (Figure 2H) (25). Moreover, modified XPC bands were observed in cells after exposure to oxaliplatin, but these appeared to be slightly less intense after exposure to cisplatin. These results confirm the importance of DDB2 in the resolution of oxaliplatin-DNA lesions (Figure 2H).

In order to determine if XPC and DBB2 both associate to the same oxaliplatin-DNA lesions, we immunoprecipitated XPC in formaldehyde-crosslinked cell lysate to capture transient and indirect protein interactions. As a control of ongoing NER activity and successful co-immunoprecipitation, we stained for GTF2H1, which is a subunit of NER factor TFIIH that is recruited by XPC following damage detection. GTF2H1 successfully co-immunoprecipitated with XPC after UV, cisplatin and oxaliplatin. In addition, an increased interaction between XPC and DDB2 30 min after UV and 2 h after oxaliplatin was observed, but not after cisplatin. This further confirms that the role of DDB2 in aiding XPC is specific for UV- and oxaliplatin-induced lesions and suggests that DDB2 is required at an early phase of damage-recognition, since it was no longer present on damaged DNA 4 and 6 h after oxaliplatin treatment (Figure 2I).

In summary, we find that in HCT116 cells, DDB2 is specifically required for the recognition of a fraction of oxaliplatin-induced DNA lesions by XPC, but not for the recognition of cisplatin-DNA lesions.

### Identification of HMGA2 in the response to platinum-DNA crosslinks

**Figure 3.**
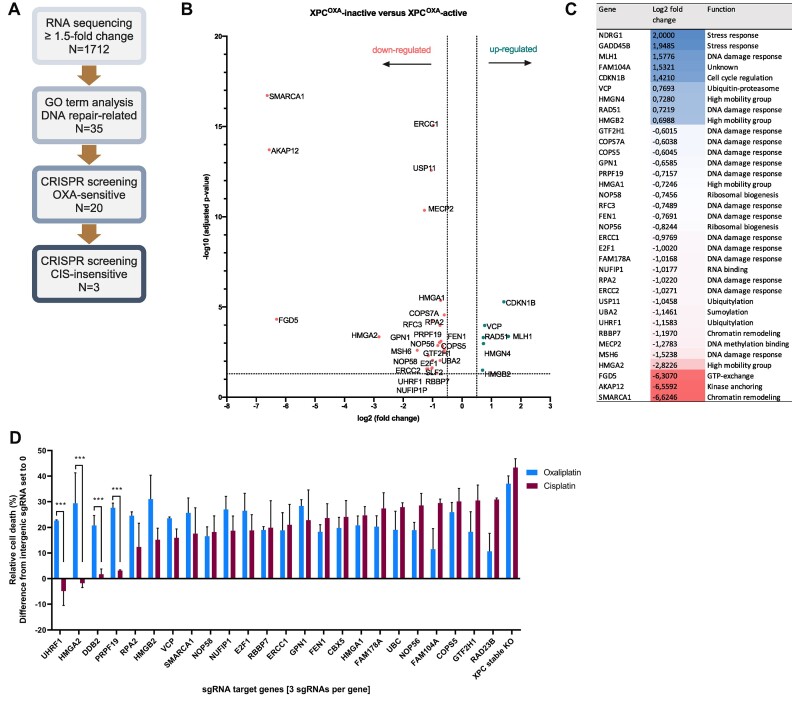
Identification of HMGA2 in the response to platinum-DNA crosslinks. (**A**) Pipeline for the selection of candidate genes potentially involved in GG-NER. The number of genes identified at each step of the analysis is given, starting by differential expression analysis by RNA sequencing, gene function analysis based on gene ontology terms and finally drug sensitivity screening. HMGA2 was selected for further validation and in-depth functional analysis. (**B**) Volcano plot showing upregulated and downregulated genes after RNA sequencing analysis of XPC^oxa^-inactive (DLD-1 and LoVo) and XPC^oxa^-active (HCT116 and SW480) cells. (**C**) Table showing the 35 genes with >1.5-fold change difference between groups that were previously functionally associated with DNA repair and chromatin-related processes and were therefore further analyzed for drug sensitivity. (**D**) Arrayed CRISPR/Cas9 drug sensitivity screen targeting genes identified in (**C**) by transduction of three pooled sgRNAs per gene in HCT116 cells. Cells were treated with 8 μM of oxaliplatin or 5 μM cisplatin and cultured for 3 days after which viability was measured using the CellTiter-Glo assay. Bars represent a difference of cell death between repair deficient and control cells (transduced with sgRNA targeting non-coding region). Graph shows a subset of 21 out of 35 genes screened for oxaliplatin and cisplatin sensitivity. XPC was included as a positive control. The full screen is shown in Supplementary Figure S5a and b. Each bar represents mean +/− SEM of three independent screens. Differences were calculated using the two-tailed t-test.

As loss of DDB2 expression does not completely abrogate XPC binding to DNA after oxaliplatin exposure, and DDB2 levels are not clearly lowered in all XPC^oxa^-inactive cells (Supplementary Figure S4b), we hypothesized that additional factors function in the recruitment of XPC to oxaliplatin-induced lesions. Therefore, we compared the expression profiles of two XPC^oxa^-inactive (DLD-1 and LoVo) and two XPC^oxa^-active (HCT116 and SW480) cell types to identify deregulated genes. We identified 1712 differently expressed genes applying a cut-off of 1.5-fold change difference between groups. Based on gene ontology annotations, we further selected genes functionally associated with chromatin-related processes, such as DNA repair and chromatin modulation which narrowed the genes down to 35 for subsequent validation (Figure 3A–C). To explore the potential involvement of the identified candidate genes in the repair of oxaliplatin-DNA lesions, we performed an arrayed CRISPR/Cas9 oxaliplatin-sensitivity screen. XPC^oxa^-active HCT116 cells were transduced with a pool of 3 sgRNAs targeting each of the 35 genes and after selection, cells were seeded for oxaliplatin sensitivity assays. Twenty out of 35 genes, together with *XPC* and *DDB2* representing positive controls, increased sensitivity to oxaliplatin upon their depletion as compared to WT cells (Supplementary Figure S5a). To further test if these genes functioned specifically in response to oxaliplatin, sensitivity to cisplatin was subsequently evaluated (Supplementary Figure S5b). As shown in Figure 3D, sgRNA-targeting the majority of genes led to hypersensitivity to both platinum drugs, suggesting that these genes are generally required for the repair of platinum-DNA crosslinks or bulky DNA lesions. Among these, several factors have not been previously associated with sensitivity to bulky DNA lesions (NOP58, NUFIP1, RBBP7, FAM178A, NOP56, FAM104A) (Figure 3D). Finally, the loss of the ubiquitin ligases UHRF1 and PRPF19, the high mobility group protein HMGA2, as well as the positive control DDB2, led to an increased sensitivity to oxaliplatin but not to cisplatin and might therefore function specifically in response to oxaliplatin-DNA lesions.

### HMGA2 is required for recognition of platinum-DNA lesions by XPC

**Figure 4.**
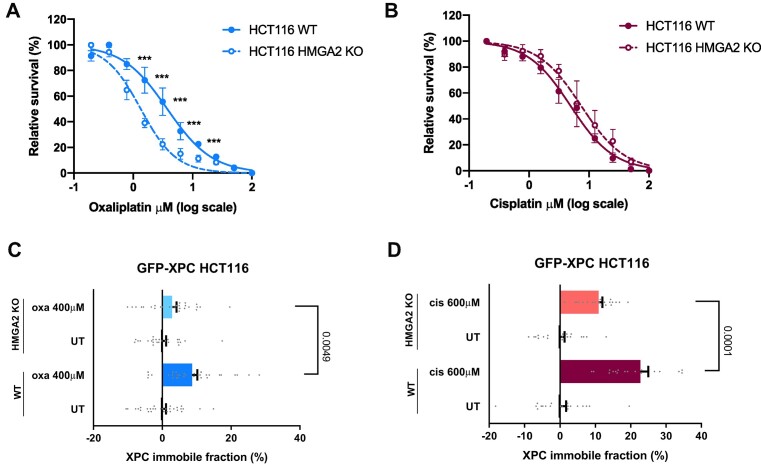
HMGA2 is required for recognition of platinum-DNA lesions by XPC. (**A**) Survival of wild type (WT) and HMGA2 KO HCT116 cells cultured for 3 days in media containing oxaliplatin, or (**B**) cisplatin. (**C**) FRAP analysis showing XPC chromatin binding in GFP-XPC KI HCT116 cells WT or HMGA2 KO treated for 6 h with 400 μM of oxaliplatin, or (**D**) with 600 μM of cisplatin. Cell viability was measured using the CellTiter-Glo assay. Each dot in the FRAP graphs represents a single cell. A minimum of 20 cells was measured per experimental condition. Unpaired two-tailed parametric *t*-test with Welch's correction without assuming a consistent standard deviation was applied for comparison of groups in FRAP the analysis. Data in (A) and (B) represent mean +/− SEM of three independent experiments. Differences were calculated by the two-tailed t-test. *** *P*-value < 0.001.

High mobility group proteins are DNA-binding proteins that alter the structure of chromatin and presumably function in DNA repair (71). Therefore, HMGA2 was selected for further validation. We stably knocked out HMGA2 by CRISPR/Cas9 in HCT116 GFP-XPC cells and confirmed protein loss by immunostaining (Supplementary Figure S6a). Next, we investigated the sensitivity of HMGA2 KO HCT116 GFP-XPC cells to both platinum drugs in a survival assay and confirmed that loss of HMGA2 results in hypersensitivity to oxaliplatin but not cisplatin (Figure 4A and B). Moreover, we observed by FRAP that loss of HMGA2 significantly decreased XPC binding to oxaliplatin-DNA lesions as well as to cisplatin-DNA lesions (Figure 4C and D). However, HMGA2 loss did not have any impact on XPC binding to UV-lesions (Supplementary Figure S6b). These results suggest that HMGA2 promotes the survival of cells exposed to oxaliplatin and the detection of both oxaliplatin- and cisplatin-lesions, but not of UV lesions, by XPC.

Thus far, the oxaliplatin and cisplatin used in our assays was aliquoted in PBS and stored at −20°C until use, taking care that both drugs did not precipitate. As aliquoting in PBS may affect the reactivity of oxaliplatin or cisplatin (72,73), we freshly prepared oxaliplatin and cisplatin in 0.9% NaCl solution and additionally tested by clonogenic survival assays whether HMGA2 loss confers oxaliplatin hypersensitivity to cells. Indeed, also with these assays and freshly prepared drugs, we found that HMGA2 is important to protect cells against oxaliplatin treatment (Supplementary Figure S6c and d). For clinical use, oxaliplatin is dissolved in 5% dextrose solution (74). To test whether this difference may influence our results, we freshly prepared oxaliplatin in 5% dextrose solution and performed again XPC-GFP FRAP experiments in DDB2 KO cells and after HMGA2 depletion by siRNA. We also tested whether simultaneous loss of DDB2 and HMGA2 reduced XPC binding to oxaliplatin-DNA lesions in an additive manner. Our results confirmed that both DDB2 and HMGA2 promote the binding of XPC to oxaliplatin lesions but did not show an additive effect (Supplementary Figure S6e). Taken together, these results suggest that, besides DDB2, more proteins, including HMGA2, modulate the ability of GG-NER to detect platinum-DNA lesions. Also, these results indicate that aliquoted oxaliplatin in PBS and freshly prepared oxaliplatin in 5% dextrose solution similarly trigger GG-NER activity.

### Low DDB2 levels predict better survival of oxaliplatin-treated colon cancer patients

**Figure 5.**
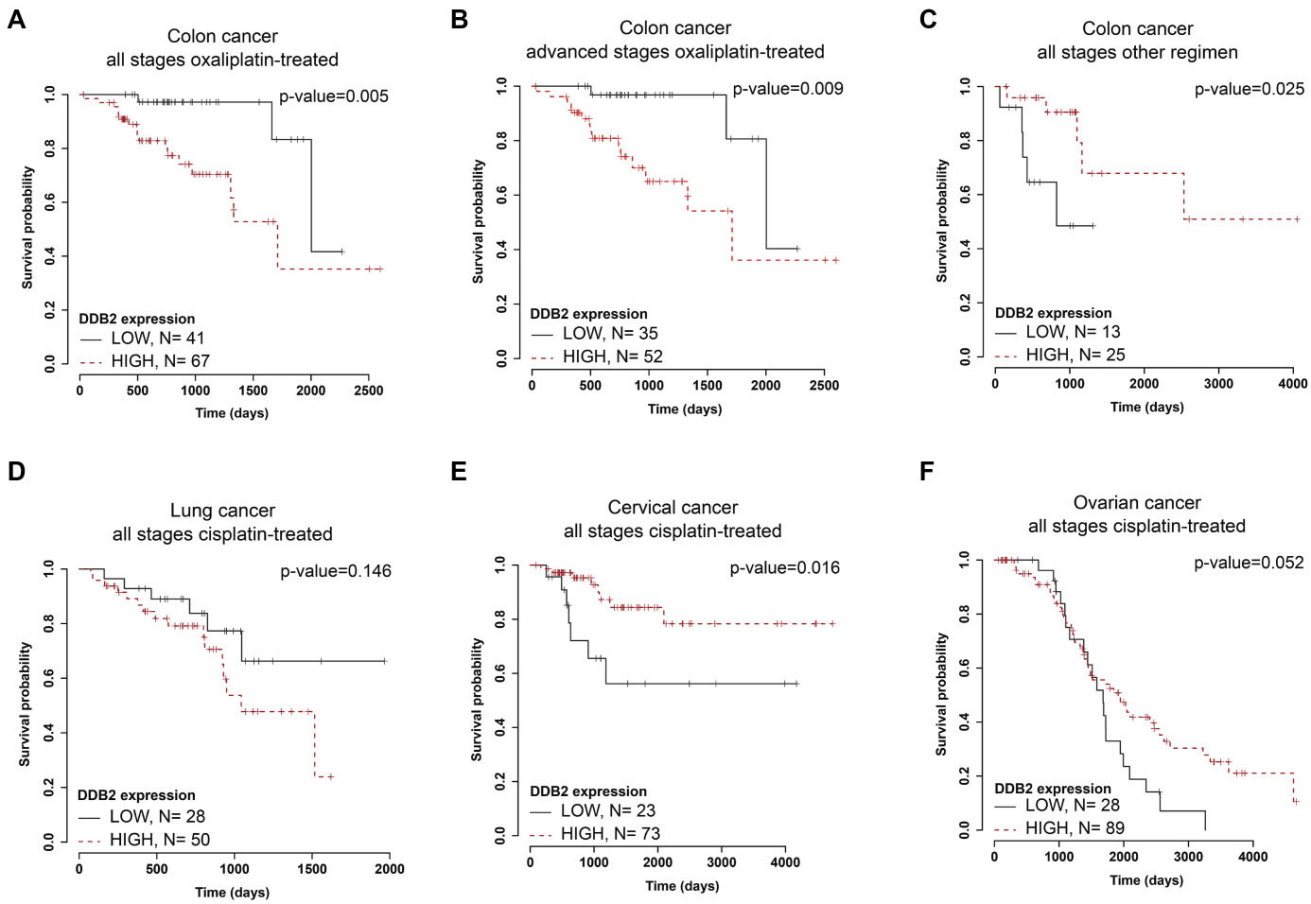
Low DDB2 levels predict better survival of oxaliplatin-treated colon cancer patients. Kaplan–Meier analysis shows association of tumor DDB2 mRNA levels with overall survival in **(A)**108 colon cancer TCGA patients clinically treated with oxaliplatin, (**B**) a subset of 87 patients diagnosed with colon cancer at TNM stage III and IV and clinically treated with oxaliplatin, and (**C**) a different group of 38 colon cancer patients clinically treated with regimens not containing oxaliplatin. (**D**) Kaplan-Meier analysis of association of tumor DDB2 expression levels with overall survival of patients diagnosed with cisplatin-treated cancers including lung cancer, (**E**) cervical cancer and (**F**) ovarian cancer. Each graph shows the mRNA level cut off leading to the best separation of the groups, the number of patients in each group and the *P*-value obtained in the logrank test.

NER is one of the major DNA repair pathways that resolves bulky adducts such as platinum-induced intrastrand crosslinks and thus dysregulation of some NER proteins has been associated with sensitivity of cancer cells to platinum drugs treatment (75). As our findings suggest that DDB2 and HMGA2 activities support the initial step of GG-NER mediated by XPC, these proteins might therefore be predictive of the clinical response to oxaliplatin. To test this hypothesis, we used the TCGA database to analyze transcriptomic data together with clinical data and overall survival outcomes from colon cancer patients. Based on the clinical data, we selected a subgroup of 108 patients for whom an oxaliplatin-containing regimen was administered. The hazard ratio from the univariate Cox regression analysis was used to identify DDR genes associated with the overall survival. Out of 2000 analyzed genes, *DDB2*, but not *XPC* nor *HMGA2*, significantly associated with hazard ratio of 2.1 in a group of oxaliplatin-treated colon cancer patients. Our analysis also revealed that higher gene expression levels of DDB2 were associated with poor overall survival. According to the Cox proportional hazards regression model, doubling DDB2 expression (FPKM) would multiply the disease life hazard by 2.1. (Supplementary Figure S7a). This association increased to a hazard ratio of 2.8 in a group of patients in advanced stage of colon cancer, with tumor-node-metastasis (TNM) stage III and IV (Supplementary Figure S7b). Kaplan-Meier analysis confirmed that DDB2 is negatively associated with overall survival (Figure 5A). Low DDB2 levels predict a significantly better survival when stratifying tumors by DDB2 expression in all 108 oxaliplatin-treated colon cancer patients irrespectively of TNM (Figure 5A and Supplementary Figure S7c). The same association held true when analyzing clinically more comparable groups of TNM III and IV patients (Figure 5B and Supplementary Figure S7d). Interestingly, the negative correlation between DDB2 expression levels and patient's overall survival was not recapitulated in the group of colon cancer patients treated with other chemotherapeutics (5-fluorouracil, leucovorin or irinotecan) (Figure 5C and Supplementary Figure S7e), suggesting that the protective effect of low DDB expression is specific to oxaliplatin treatment. Moreover, we did not observe any negative association between DDB2 and overall survival in other cancers routinely treated with cisplatin, including ovarian, lung, and cervical cancer (Figure 5D–F).

**Figure 6.**
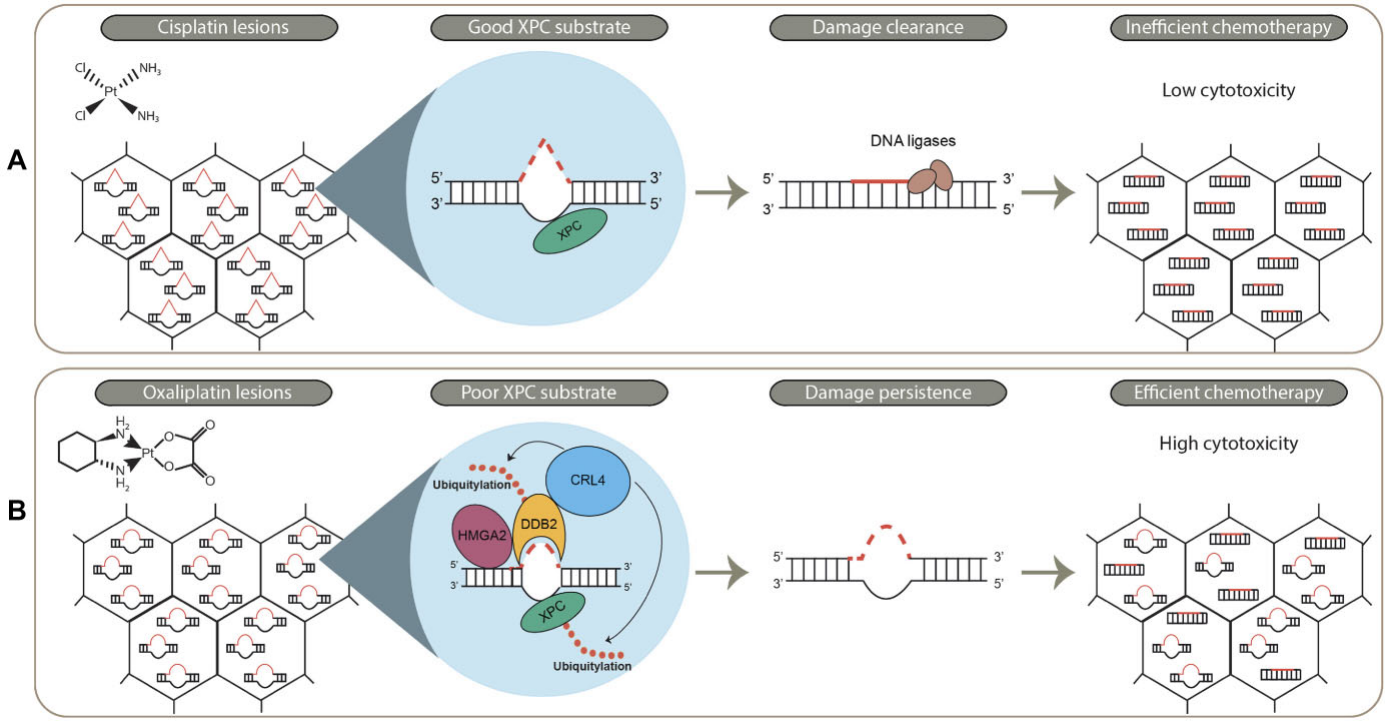
Removal of oxaliplatin- and cisplatin-DNA lesions requires different global genome repair mechanisms that affect their clinical efficacy. Cisplatin and oxaliplatin are commonly used anti-cancer chemotherapeutics due to their ability to generate bulky DNA lesions. The different structure of the lesions requires distinct DNA repair mechanisms. (**A**) Structural distortions of cisplatin-DNA lesions enable XPC to efficiently recognize and bind the lesion site and therefore to activate GG-NER. Cisplatin-DNA lesions are a good substrate for XPC, which compromises the cytotoxicity of cisplatin in GG-NER proficient tumors. (**B**) Structural distortions of oxaliplatin-DNA lesions are inefficiently recognized and bound by XPC. XPC therefore requires DDB2 and HMGA2 for efficient GG-NER initiation and thus reduced expression leads to inefficient damage recognition. Oxaliplatin-DNA lesions thus are poor XPC substrates, which leads to higher cytotoxicity of the drug. Low DDB2 expression levels in tumors therefore predict a favorable clinical outcome of oxaliplatin-treated patients.

Taken together, we show that low DDB2 levels associate with better overall survival of colon cancer patients treated with oxaliplatin, which, based on our analysis of GG-NER in different colon cancer cell lines, might be due to less efficient detection of oxaliplatin-DNA lesions by GG-NER. Low DDB2 levels therefore likely represent a specific predictive marker for oxaliplatin treatment in colon cancer (Figure 6).

## Discussion

Platinum drugs represent first-line conventional therapy for most solid tumors. Their high affinity for DNA, resulting in formation of bulky DNA lesions that subsequently inhibit essential cellular processes such as transcription and replication, make them effective anti-cancer drugs (76). Oxaliplatin is an efficient alternative treatment for cisplatin-refractory cancers, such as colon and rectal cancer and other gastrointestinal cancers. Due to its bifunctional DNA binding, oxaliplatin induces both intrastrand crosslinks and ICLs (9), whose resolution requires multiple different DNA repair pathways. Indeed, previously, we found that disruption of multiple major DNA repair pathways leads to hypersensitivity of colon cancer cells to oxaliplatin (Supplementary Figure S1a and (16). Strikingly, the only exception was GG-NER, as impairment of XPC or DDB2 had no impact on the sensitivity of DLD-1 colon cancer cells to oxaliplatin, while it did cause hypersensitivity to cisplatin. These results suggest that the structure of DNA lesions induced by oxaliplatin and cisplatin is significantly different and that their removal requires different DNA repair mechanisms. Oxaliplatin crosslinks might be less helix-distorting than cisplatin crosslinks and therefore poor substrates for XPC binding. Indeed, oxaliplatin and cisplatin adducts were shown to induce DNA conformation differences that are potentially related to the differential ability of DNA repair proteins to discriminate between them (77) and oxaliplatin-DNA lesions were shown to cause less distortion of DNA than cisplatin-DNA lesions (78).

In this study, we show that a subgroup of colon cancer cells is intrinsically deficient in the removal of oxaliplatin lesions by GG-NER, which might explain why oxaliplatin is superior to cisplatin in colon cancer treatment. As oxaliplatin mainly induces 1,2- and 1,3-intrastrand crosslinks (9), and these are the type of lesions removed by GG-NER (15,61,65), it is very probable that XPC does not efficiently recognize these oxaliplatin-induced intrastrand crosslinks in these cell lines. It is unlikely that XPC itself is defective, because deletion of XPC still caused cisplatin sensitivity in all cells, while ectopically expressed XPC-GFP still did not bind efficiently to oxaliplatin-DNA lesions in DLD-1 cells. DDB2 is well known to promote the binding of XPC to UV-induced CPD lesions that are not sufficiently DNA helix distorting to be recognized by XPC alone (24–26). Here, we provide evidence that DDB2 also promotes XPC binding to oxaliplatin-DNA lesions, but not to cisplatin-DNA lesions. A lower expression of DDB2, as observed in some of the XPC^oxa^-inactive cells, can therefore at least partially explain why some colon cancer cells lack an effective GG-NER response to oxaliplatin-induced intrastrand crosslinks.

Since loss of DDB2 only partially impaired the recognition of oxaliplatin-DNA lesions by XPC, additional factors probably exist that regulate recognition of intrastrand crosslinks. Indeed, multiple chromatin remodeling factors have previously been implicated in facilitating the recognition of UV lesions by XPC, but their relevance to recognition of platinum-DNA lesions is unknown (30). Here, through gene expression analysis, we identified HMGA2 as an important novel regulator of the recognition and repair of oxaliplatin-lesions via XPC and GG-NER. Whether HMGA2 is similarly important for the repair of cisplatin-DNA lesions is at this stage unclear, as HMGA2 promoted XPC binding to cisplatin-DNA lesion but did not strongly affect cellular survival after cisplatin exposure. HMGA2 is a small AT-hook DNA binding protein that is thought to drive tumorigenesis in many types of cancers by affecting proliferation, epithelial-to-mesenchymal transition, apoptosis, DNA repair and other processes (79,80). There is a body of evidence that proposes a role for HMG proteins like HMGA2 in DNA repair as well as in cancer pathogenesis and treatment (81,82). Notably, HMGB1 and HMGB4 proteins have been proposed to sensitize cells to cisplatin by binding to cisplatin-DNA lesions and preventing these from being repaired by NER (7,83–85). Interestingly, HMGA2 has also been implicated in regulating NER, by binding to the ERCC1 promoter (86), but our results suggest that HMGA2 directly functions in NER by promoting the recognition of oxaliplatin-induced intrastrand crosslinks. As HMGA2 depletion did not further reduce XPC binding to oxaliplatin-DNA lesions in DDB2 KO cells, HMGA2 and DDB2 probably act in the same pathway. It will therefore be interesting to test if HMGA2 affects DDB2 binding and/or whether HMGA2 itself binds to oxaliplatin-DNA lesions. Also, whether it is HMGA2 chromatin remodeling activity (87) or its 5′-deoxyribosyl phosphate (dRP) lyase and apurinic/apyrimidinic (AP) lyase activity (88) that is utilized in GG-NER remains to be understood. In line with our findings, HMGA2 has been reported to be upregulated in oxaliplatin-resistant colorectal cancer, thus further strengthening the findings that HMGA2 functions in the clearance of DNA lesions induced by oxaliplatin (89).

There are major drawbacks to using platinum drug therapy, because of the severe side effects and because many tumors do not respond to treatment and those that do, often develop therapy resistance (90–92). The lack of predictive biomarkers for tumor sensitivity makes patient stratification for platinum-based chemotherapeutics problematic. We postulated that lower GG-NER activity in colon cancer, due to intrinsically low levels of any of the DDR factors, could lead to accumulation of unrepaired DNA damage and therefore more efficient treatment. Indeed, we found that low DDB2 levels are significantly associated with higher survival probability in oxaliplatin-treated colon cancer patients. This association was not confirmed in colon cancer patients treated with other regimens, or in other types of cancers treated with cisplatin. Importantly, early reports have shown that DDB2 mRNA levels correlate well with DDB2 protein levels and DNA damage binding activity in cells (26,93,94). Therefore, DDB2 levels appear to be a valuable predictive marker, specifically for the response to oxaliplatin, and could be a beneficial biomarker for treatment selection which could have important implications for the management of colon cancer. Strikingly, we did not find an association of HMGA2 expression levels with survival in oxaliplatin-treated colon cancer patients, while we initially identified HMGA2 because it was differentially regulated between XPC^oxa^-active and inactive cells. This likely indicates that HMGA2 expression levels *in vivo* do not vary to such an extent between cells as to strongly influence GG-NER activity.

It should be noted that for the majority of our experiments, we used oxaliplatin and cisplatin dissolved and aliquoted in PBS, while for use in the clinic cisplatin is dissolved in salt and oxaliplatin in dextrose solution. Upon clinical administration, once in inside the cell, cisplatin undergoes hydrolysis and oxaliplatin undergoes chlorination and hydrolysis, forming the final forms of the drugs that react with DNA (95). Chlorination of oxaliplatin already happens in PBS (72,73), which may therefore affect the chemical properties of oxaliplatin and the interpretation of our results. To ensure that our identification of DDB2 and HMGA2 as specific regulators of the oxaliplatin response was not due to changed reactivities of oxaliplatin and cisplatin in PBS, we confirmed their involvement by performing FRAP and survival experiments with freshly prepared oxaliplatin and cisplatin, in salt and dextrose solution. In conclusion, our findings provide new mechanistic insights into the clearance of intrastrand crosslink lesions induced by oxaliplatin, by assigning dedicated functions for DDB2 and HMGA2 in GG-NER, and by the identification of DDB2 as new biomarker that could be used for treatment stratification.

## Supplementary Material

zcad057_supplemental_file

## Data Availability

The data discussed in this publication have been deposited in NCBI’s Gene Expression Omnibus (96) and are accessible through GEO Series accession number GSE227871 (https://www.ncbi.nlm.nih.gov/geo/query/acc.cgi?acc=GSE227871). Supporting data are available in online supplementary material.
