# Supplementary material for: Detection of oxaliplatin- and cisplatin-DNA lesions requires different global genome repair mechanisms that affect their clinical efficacy
Source: NAR Cancer. 2023 Dec 5;5(4):zcad057. doi: 10.1093/narcan/zcad057 (PMC10696645; doi:10.1093/narcan/zcad057)
Supplement: zcad057_supplemental_file [file zcad057_supplemental_file.pdf]

Supplementary data

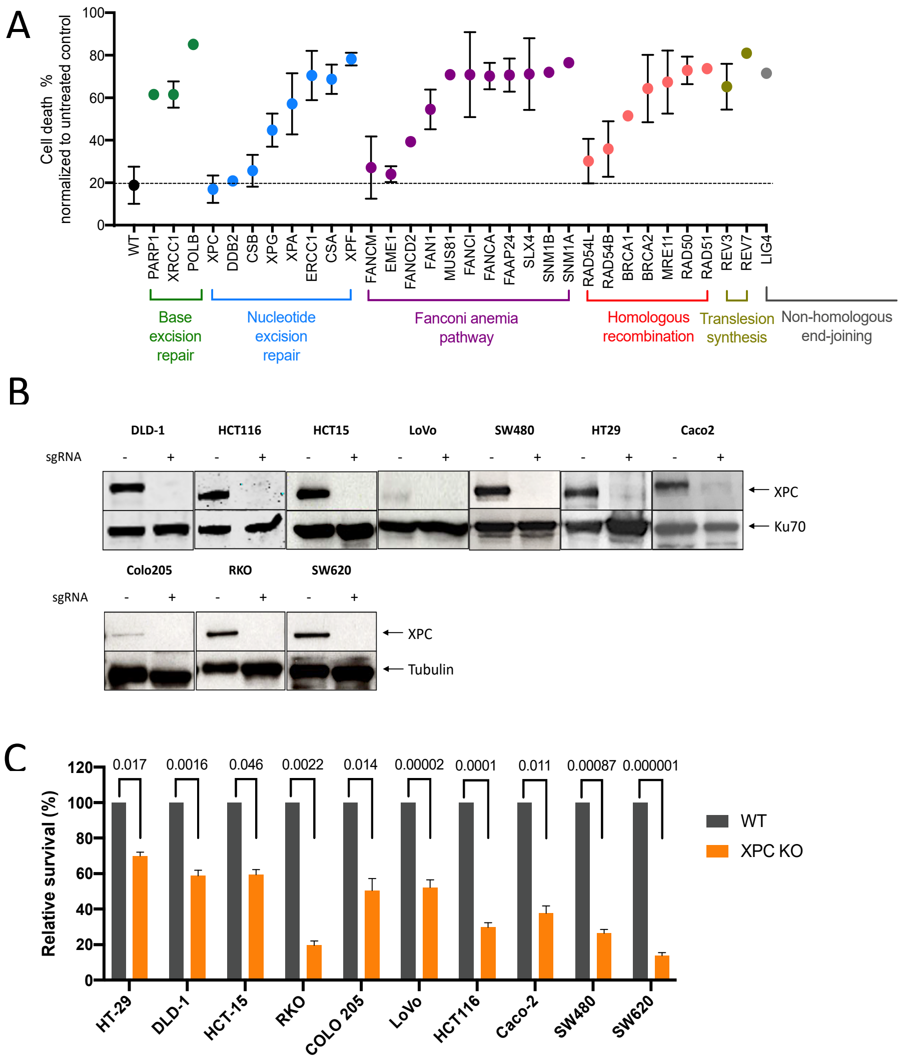

Supplementary Figure S1. (A) DDR-focused CRISPR/Cas9 screen for genes whose depletion leads to oxaliplatin sensitivity in DLD-1 cells. Cells were transduced with an sgRNA targeting each gene, clonally expanded and treated with 15 µM of oxaliplatin. Cell were cultured for 3 days after which their viability was measured using the MTT assay. Each data point represents the mean +/- SD of two independent screens (adapted from (15)). **(B)** Immunoblot analysis showing CRISPR/Cas9-generated XPC KO in 10 colon cancer cell lines. Ku70 or tubulin was used as loading control. **(C)** Sensitivity of WT and XPC KO colon cancer cells to 4 J/m^2 UV. Relative survival of XPC KO cells is shown as a percentage survival of their WT counterparts. Bars represent mean +/- SEM of 3 independent experiments. Differences were calculated using the two-tailed t-test.

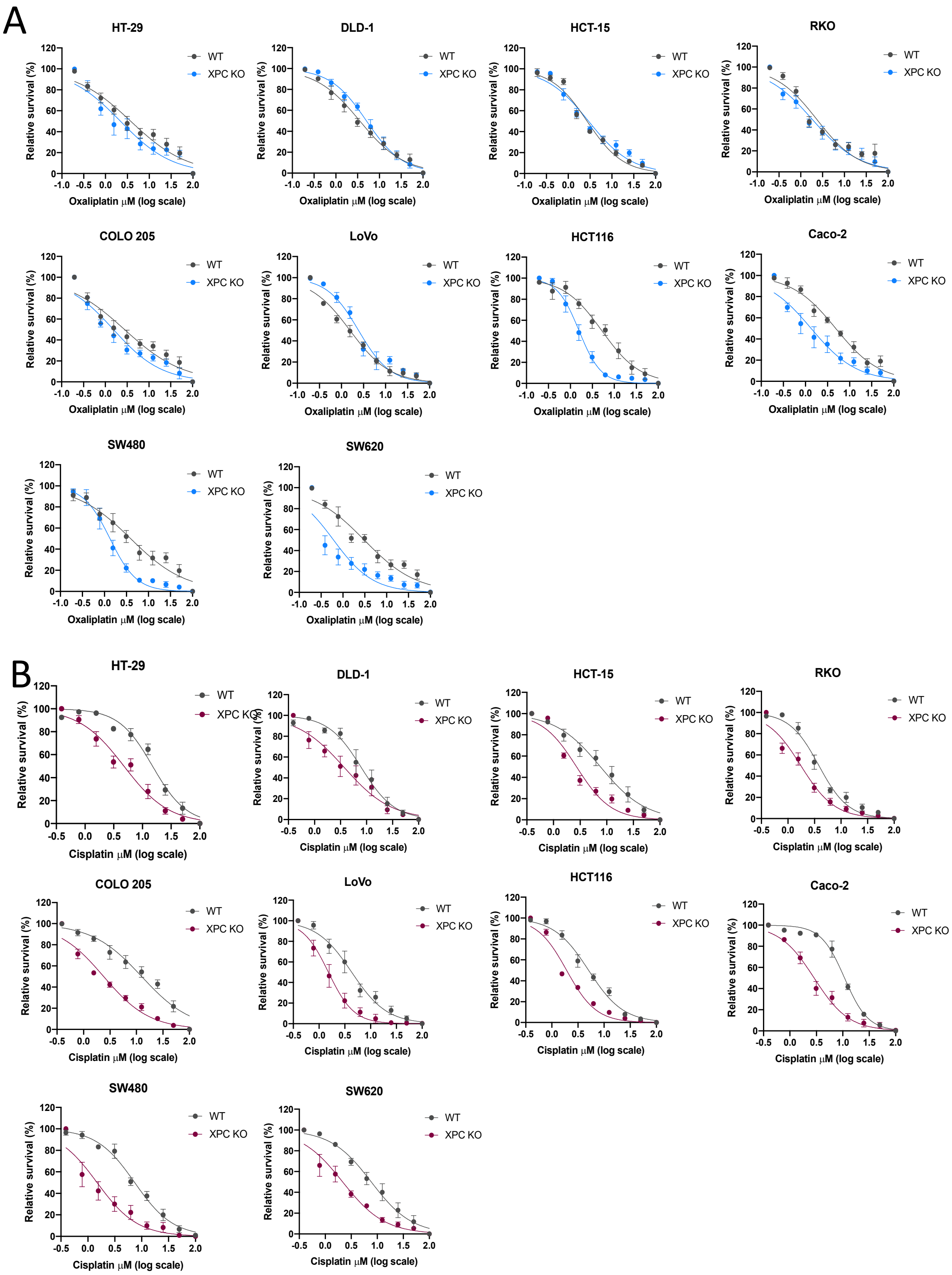

Supplementary Figure S2. (A) Dose-response curves of WT and XPC KO colon cancer cells to oxaliplatin and **(B)** cisplatin. These dose-response curves were used to calculate LD50 concentrations for each cell line and each drug presented in Figure 1A-1D. Each data point represents mean +/- SEM of 3 independent experiments.

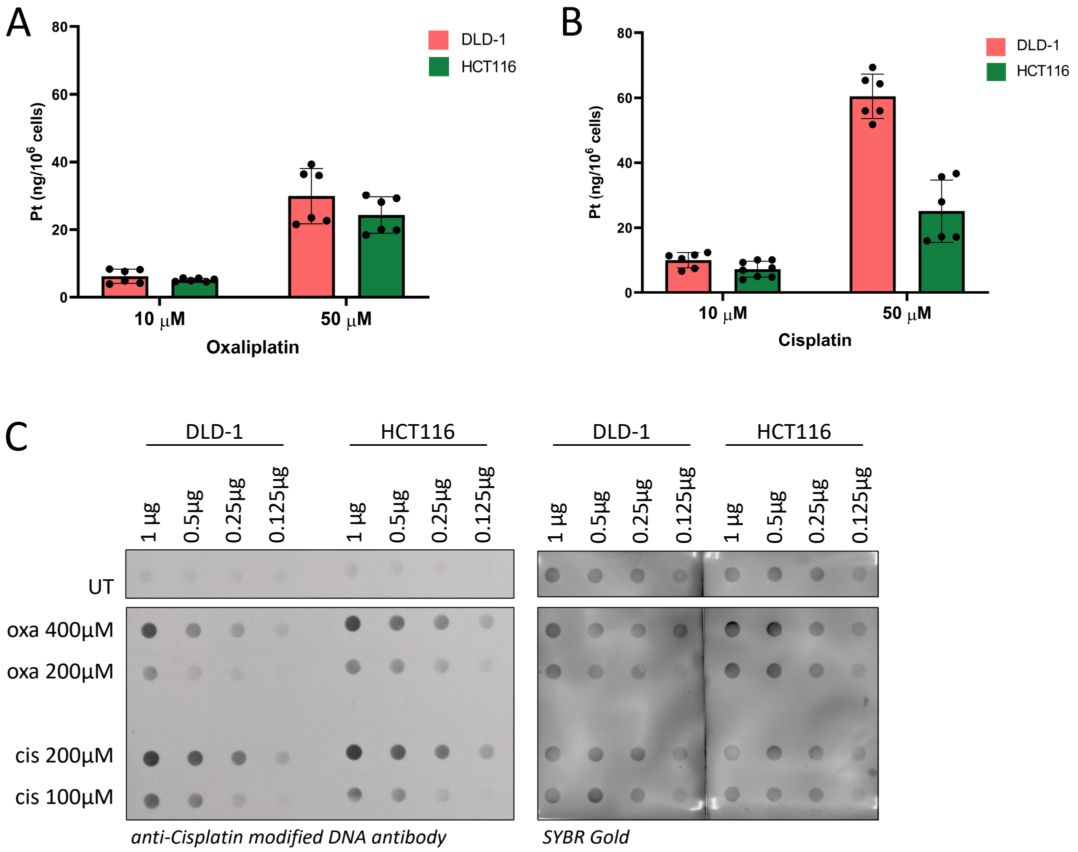

Supplementary Figure S3. (A) Cellular platinum uptake measured by ICP-MS in DLD-1 and HCT116 cells treated with 10 μM or 50 μM of oxaliplatin or **(B)** cisplatin for 6 h. Bars represent mean +/- SD of 2 independent experiments each performed in technical triplicate. **(C)** Dot blot showing staining of platinum-DNA crosslinks by an anti-cisplatin DNA modified antibody. DLD-1 and HCT116 cells were treated for 6 h with the indicated concentrations of oxaliplatin and cisplatin, after which DNA was isolated and transferred onto the membrane in the indicated quantities. Staining with SYBR Gold was used as a loading control.

A

Group	Cell line	MS status	MMR gene dysfunction	TP53 status	CIMP	CIN	KRAS	BRAF	PIK3CA	PTEN
XPC^{oxa}-active	HCT116	MSI	MLH1	WT	+	-	G13D	WT	H1047R	WT
	SW480	MSS	N.A.	R273H; P309S	-	+	G12V	WT	WT	WT
	SW620	MSS	N.A.	R273H; P309S	-	+	G12V	WT	WT	WT
	Caco2	MSS	N.A.	E204X	-	+	WT	WT	WT	WT
XPC^{oxa}-inactive	DLD-1	MSI	MSH6	S241F	+	-	G13D	WT	E545K; D549N	WT
	LoVo	MSI	MSH2	WT	-	-	G13D; A14V	WT	WT	WT
	HT29	MSS	N.A.	R273H	+	+	WT	V600E; T119S	P449T	WT
	Colo205	MSS	N.A.	Y107fs; Y103fs	+	+	WT	V600E	WT	WT
	RKO	MSI	MLH1	WT	+	-	WT	V600E	H1047R	WT
	HCT15	MSI	MSH6	S241F	+	-	G13D	WT	E545K; D549N	WT

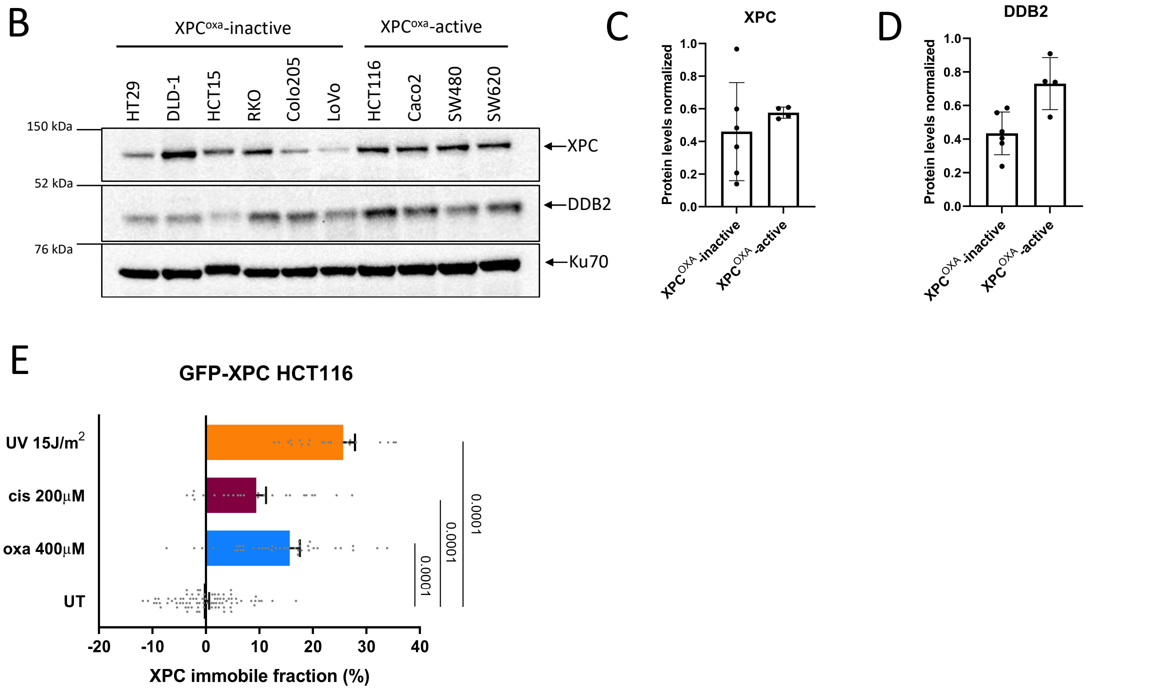

Supplementary Figure S4. (A) Table showing genetic characteristics of ten colon cancer cell lines as reported in the literature (63). MSI, microsatellite instable; MSS, microsatellite stable; MMR, mismatch repair; CIMP, CpG island methylator phenotype; CIN, chromosomal instability. **(B)** Immunoblot analysis and quantification **(C, D)** of XPC and DDB2 protein levels in lysates of ten colon cancer cell lines. Ku70 was stained as a loading control. **(E)** FRAP analysis showing XPC immobilization in GFP-XPC KI HCT116 cells treated with indicated concentrations of oxaliplatin or cisplatin for 6 h or irradiated with 15 J/m^2 UV. Each dot in the FRAP graphs represents a single cell. A minimum of 24 cells was measured per experimental condition. Unpaired two-tailed parametric t-test with Welch's correction without assuming a consistent standard deviation was applied for comparison of groups in the FRAP analysis.

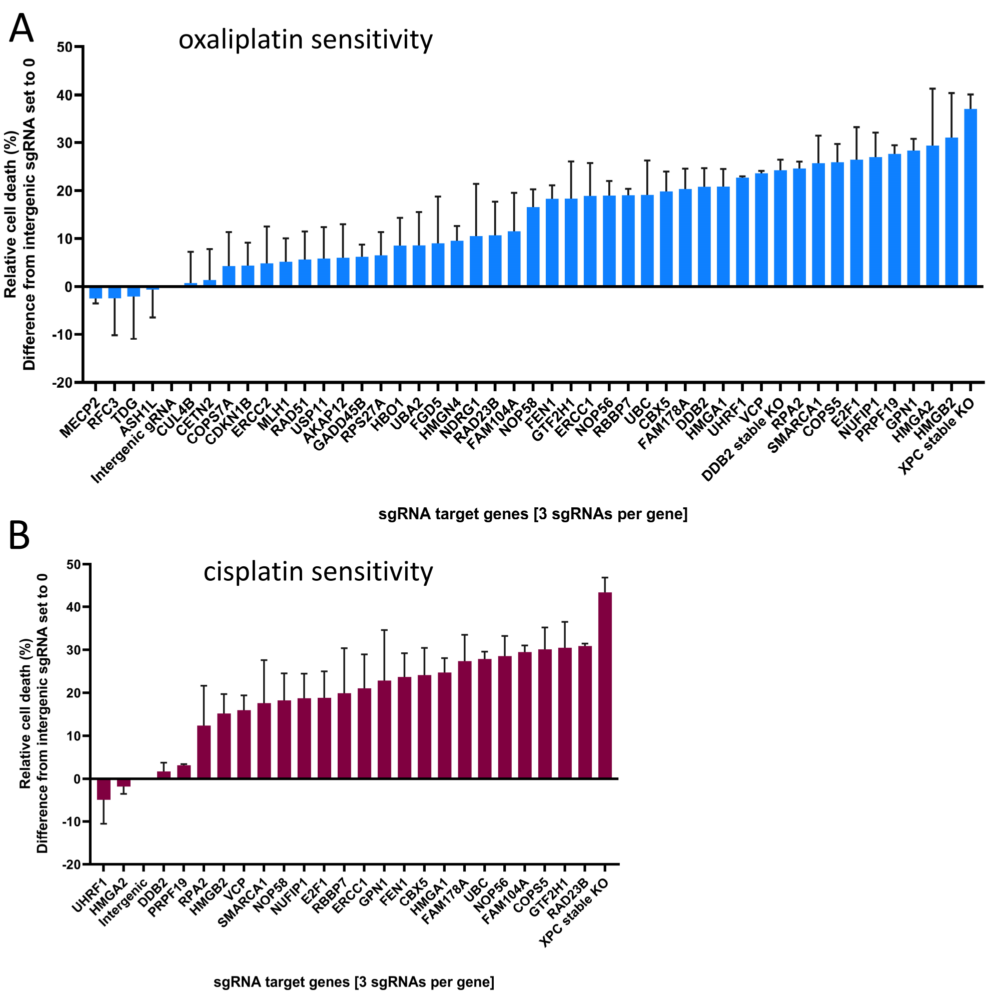

Supplementary Figure S5. (A) Arrayed CRISPR/Cas9 drug sensitivity screen targeting 35 candidate genes by transduction of 3 pooled sgRNAs per gene in HCT116 cells. Cells were treated with 8 µM of oxaliplatin for 3 days and viability was measured by CellTiter-Glo assay. Bars represent a difference of cell death between cells transduced with the sgRNAs targetting DNA damage response genes and cells transuced with the sgRNA targeting a non-coding region. XPC and DDB2 stable KO HCT116 cells were used as positive controls. HCT116 cells transduced with an sgRNA targeting a non-coding region was used as a negative control. **(B)** Twenty-one genes that showed sensitivity to oxaliplatin were analyzed for sensitivity to 5 µM of cisplatin in the same experimental setting as described in (A). A subset of this data is also presented in Figure 3D. Bars represent mean +/- SEM of 3 independent screens.

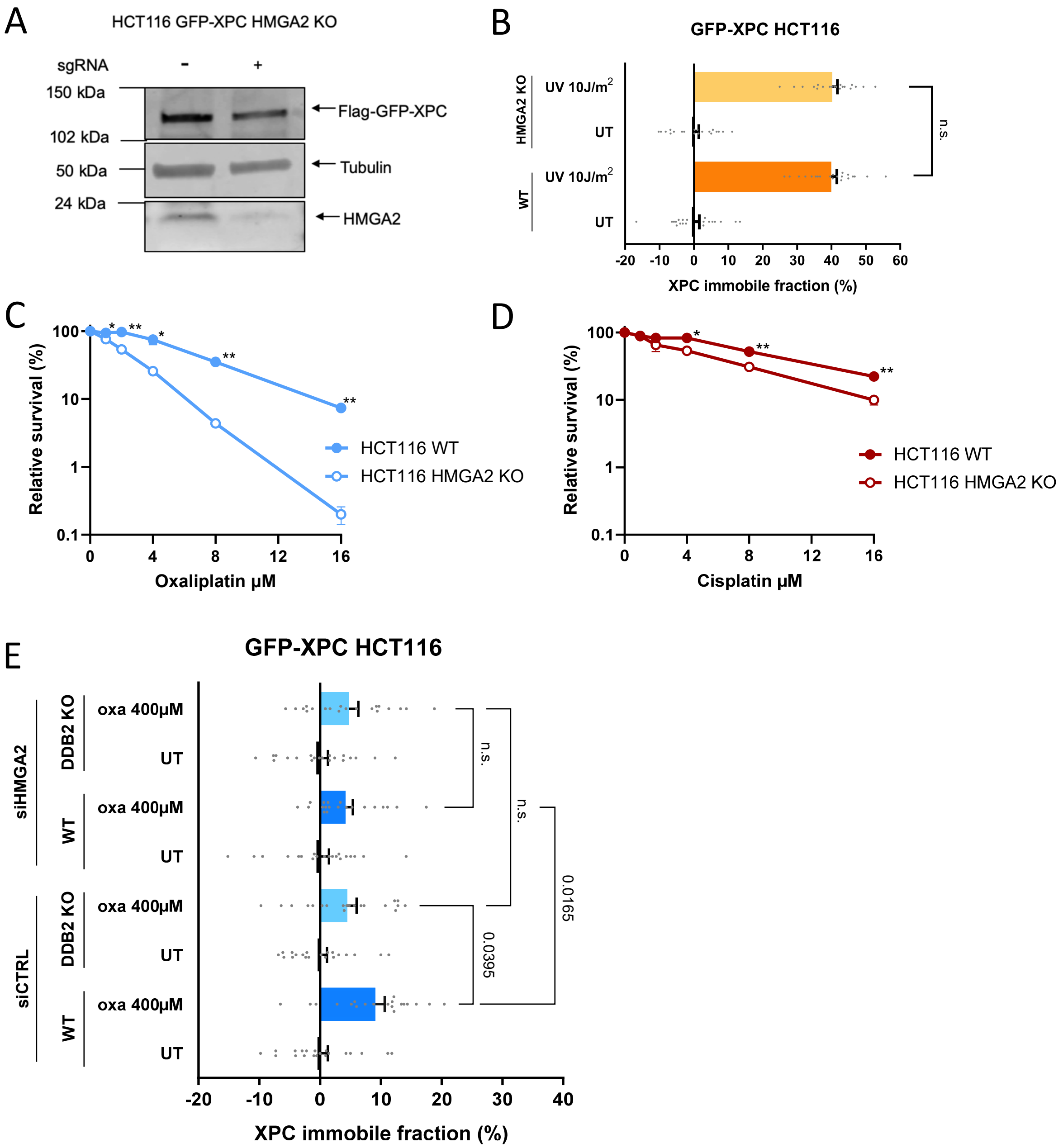

Supplementary Figure S6. (A) Immunoblot analysis of HMGA2 protein levels in WT and HMGA2 KO GFP-XPC KI HCT116 cells. Tubulin was stained as a loading control **(B)** FRAP analysis showing XPC immobilization in HMGA2 WT or KO GFP-XPC HCT116 cells irradiated with 10 J/m^2 UV. Each dot in the FRAP graphs represents a single cell. A minimum of 20 cells was measured per experimental condition. Unpaired two-tailed parametric t-test with Welch's correction without assuming a consistent standard deviation was used for comparison of the groups in the FRAP analysis. **(C)** Oxaliplatin clonogenic survival assay of WT and HMGA2 KO HCT116 cells. **(D)** Cisplatin clonogenic survival assay of WT and HMGA2 KO HCT116 cells. **(E)** FRAP analysis showing XPC chromatin binding in WT and DDB2 KO GFP-XPC KI HCT116 cells treated with control siRNA (siCtrl) or siRNA targeting HMGA2 (siHMGA2), either untreated (UT) or treated for 6 h with 400 μM of oxaliplatin. Each dot in the FRAP graphs represents a single cell. 20 cells were measured per experimental condition. Unpaired two-tailed parametric t-test with Welch's correction without assuming a consistent standard deviation was applied for comparison of groups in the FRAP analysis. Data in C and D represent mean +/- SEM of 3 independent experiments. Differences were calculated using the two-tailed t-test. * p-value < 0.05 ** p-value < 0.01.

A

Gene	p.val Cox	HR
CEBPG	0.025	0.27
DYNLT3	0.027	0.48
EI24	0.0064	0.29
EPC1	0.043	3.8
ERCC4	0.038	3
NUDT15	0.0039	0.4
PERP	0.037	0.49
RAB2A	0.035	0.39
SBDS	0.021	0.46
TOP1	0.02	0.45
UBA2	0.037	0.38
UCHL3	0.0043	0.38
DMTF1	0.034	2.4
MDM4	0.029	2.2
METTL3	0.01	3.7
RAD52	0.029	2.1
SENP5	0.041	3.7
CDK9	0.0031	6.5
CLK2	0.016	2.5
TSPYL2	0.014	2.6
TUBG2	0.031	2.2
BCL7C	0.043	0.33
DDB2	0.014	2.1
ING4	0.033	3.1
POLR2H	0.024	3.8
PPP2R2D	0.033	4.3
ZBTB3	0.036	2.5
CTC1	0.0057	3.5
EIF4A1	0.0083	2.6
GPS2	0.028	2.2
TINF2	0.034	3.8
TPP1	0.028	2.7
TSC1	0.043	2.8
ZFYVE26	0.048	2.6
DDX11	0.0089	2.8
FBH1	0.00067	15
ADARB1	0.018	2.2
ANKRD44	0.017	2.2
DTX1	0.00055	2.5
FBXO10	0.047	2.3
NOTCH4	0.0081	2.3
NUAK1	0.0038	2.1
PTPN6	0.0034	6.4
TCF7L1	0.013	2
VASH1	0.0033	2.5
ANAPC2	0.003	3.4
CUL7	0.019	2.4
CUL9	0.016	2
ENDOV	0.0031	3
NISCH	0.026	2.7
PHF1	0.034	2.6
TUBGCP6	0.015	2.3
UBA7	0.028	2.3
ALDH4A1	0.0066	0.49
AURKA	0.014	0.45
CDC25C	0.016	0.45
DPY30	0.046	0.4
GPI	0.017	0.3
PDCD2L	0.031	0.46
VDAC1	0.0016	0.22
YBX1	0.046	0.38
DAXX	0.034	5
RING1	0.034	3.1

B

Gene	p.value in advanced desease	HR in advanced desease
CEBPG	0.032	0.26
DYNLT3	0.018	0.44
EI24	0.0023	0.25
EPC1	0.016	5.7
ERCC4	0.071	2.7
NUDT15	0.0081	0.43
PERP	0.074	0.53
RAB2A	0.032	0.37
SBDS	0.046	0.48
TOP1	0.037	0.47
UBA2	0.055	0.39
UCHL3	0.011	0.41
DMTF1	0.012	3
MDM4	0.014	2.9
METTL3	0.0057	4.8
RAD52	0.0026	2.8
SENP5	0.042	3.9
CDK9	0.0053	6.4
CLK2	0.0084	3.1
TSPYL2	0.0044	3.6
TUBG2	0.024	2.3
BCL7C	0.02	0.27
DDB2	0.019	2.8
ING4	0.0086	4.3
POLR2H	0.014	5.2
PPP2R2D	0.039	4.6
ZBTB3	0.037	2.7
CTC1	0.0014	5.1
EIF4A1	0.0013	3.9
GPS2	0.018	2.6
TINF2	0.18	2.4
TPP1	0.089	2.3
TSC1	0.015	4
ZFYVE26	0.18	1.9
DDX11	0.00054	4.2
FBH1	4.8e-05	27
ADARB1	0.059	1.9
ANKRD44	0.064	1.9
DTX1	0.0015	2.4
FBXO10	0.088	2.2
NOTCH4	0.01	2.3
NUAK1	0.026	1.8
PTPN6	0.0042	8.3
TCF7L1	0.017	2
VASH1	0.0088	2.3
ANAPC2	0.00073	4.5
CUL7	0.0038	3.7
CUL9	0.0096	2.2
ENDOV	0.0066	2.8
NISCH	0.013	3.3
PHF1	0.065	2.3
TUBGCP6	8e-04	3.9
UBA7	0.062	2.3
ALDH4A1	0.015	0.5
AURKA	0.021	0.46
CDC25C	0.0056	0.39
DPY30	0.046	0.33
GPI	0.0067	0.26
PDCD2L	0.041	0.48
VDAC1	0.016	0.27
YBX1	0.088	0.42
DAXX	0.031	5.4
RING1	0.026	3.5

C

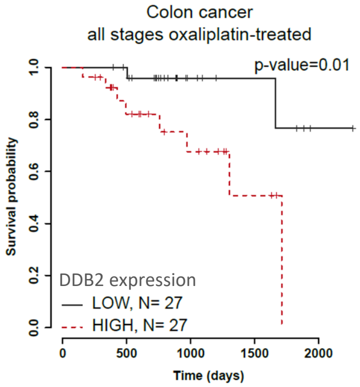

D

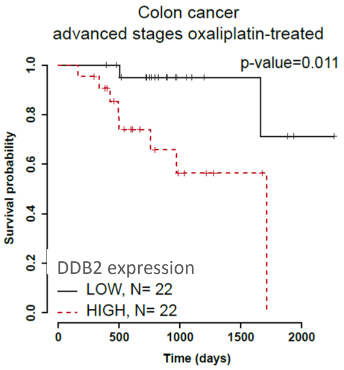

E

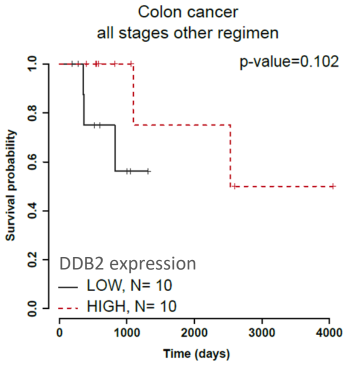

Supplementary Figure S7. (A) Univariate Cox regression analysis for calculation of hazard ratios (HR) of 2,000 DNA damage response genes identifies DDB2 as being significantly associated with overall survival of oxaliplatin-treated colon cancer patients and **(B)** patients diagnosed in the advanced stages with TNM III and IV. **(C)** Kaplan-Meier analysis shows association of tumor DDB2 mRNA levels with overall survival in 54 TCGA colon cancer patients clinically treated with oxaliplatin. Figure shows data from the same patients' group as in Figure 5A but compares patients in lower versus upper mRNA levels quantiles. **(D)** A subset of 44 patients diagnosed with colon cancer in TNM stage III and IV and clinically treated with oxaliplatin. Figure shows a subgroup of patients from Figure 5B, comparing patients in lower versus upper mRNA levels quantiles. **(E)** An independent group of 20 colon cancer patients clinically treated with regimens not containing oxaliplatin. Figure is associated to Figure 5C but compares lower versus upper mRNA levels quantiles. Each graph shows the number of patients in each group and the p-value.

Table S1. Sequence of sgRNAs used for generation of XPC KO colon cancer cells and the primers for the amplification of sgRNA-target site used for subsequent verification of indels.

Gene	SgRNA	SgRNA sequence	PCR primer forward	PCR primer reverse	Cell line
XPC	#1	GCTCGGAAACGCGCGGCCGG	TTCCTTTAGGGGCGTGACTA	ACTGCCTTCTCCGAGTTCAA	DLD-1<br>HCT116<br>SW620<br>Caco-2<br>COLO 205
XPC	#2	GGACCATCTGCTGAACCCCC	GTGCTTGAGGACAGTAGTATGAGAT	CTCACCTTATGTTCTGTGTTGTCAC	LoVo<br>SW480<br>RKO<br>HCT15<br>HT29

Table S2. Sequence of sgRNAs used for generation of stable KOs of candidate genes in GFP-XPC KI HCT116 cells. Primers used for amplification of sgRNA-target sites prior to verification of indel frequency by sanger sequencing and TIDE.

Gene	Gene ID Ensembl	SgRNA	SgRNA sequence	PCR primer forward	PCR primer reverse
HMGA2	ENSG00000149948	#1	ACTGGAGAAAAACGGCCAAG	CGATACGTCATCTGCAAAGC	CACCCATTTCCTAGGTCTGC
		#2	AAGAGGCAGACCTAGGAAAT	AGAAAGCAGAAGCCACTGGA	CCTGGAGCCTAGTGGTAGGA
DDB2	ENSG00000134574	#1	GGTATCGGCCCACAACAATG	TTACAGGTGTGAGCCACTGC	ACGTCGATCGTCCTCAATTC
		#2	GGGGCGTAATACAATCTCGG	CCTCTCAATCCTCCCTCCAT	TATTCAAGCAGCAGGCACAG

Table S3. SgRNA sequences complementary to 35 candidate genes, *DDB2* and a non-coding region used as a negative control. SgRNAs were designed to deplete gene products in HCT116 cells in an arrayed knock-out sensitivity CRISPR/Cas9 screen.

Gene	Gene ID Ensembl	SgRNA #1	SgRNA #2	SgRNA #3
HMGA2	ENSG00000149948	GCAGCCGTCCACTTCAGCCC	CAAGAGGCAGACCTAGGAAA	AAGTCAGTACGAGGGCGCGG
HMGA1	ENSG00000137309	ACCCGGGTGAGACTTGAGAT	CTCAAGTCTCACCCGGGTCT	GACCCGGGTGAGACTTGAGA
HMGB2	ENSG00000164104	GAACGAACCAGAAGATGAGG	TACGAGGACATTTTGCCCCG	TGGACGCGGATCTGTCAACA
HMGN4	ENSG00000182952	AACCGAGCTGATCTCCTCTG	ATGGTAGCAGCAGTGTTCGC	AGGCATCTCGGTTTTTTGCA
MSH6	ENSG00000116062	TCAAAGGAAGCCCAGAAGGG	GATCTGTAGGATCATTACCA	AAGGCGAAGAACCTCAACGG
FAM178A/SLF2	ENSG00000119906	AAAGAGAACAGAGAGTCCTG	AAGCCTCCTGCTCTTTCCAA	GAAGACATAAGGCAAGGCCG
E2F1	ENSG00000101412	TTTCGCCACAGGTGAAGCGG	TGAGACCCAGCTCCAAGCCG	GAGGCCGAAGTGGTAGTCGA
SMARCA1	ENSG00000102038	GATAGTGGCGGTCGCATCCG	AAACAAATGACACGGAGAGA	GCTGACAATATGCTCATCAG
RBBP7	ENSG00000102054	TGATGAGCAGAATCATCTGG	TTAAGAGGTCACCAGAAGGA	TGTCTGTGGGATATAAACGC
UHRF1	ENSG00000276043	GCGGGGCTTCTGGTACGACG	GCGGACCTCGTAGTCGAAGA	GGTACGACGCGGAGATCTCC
NUFIP1	ENSG00000083635	ATTTCGAGACTCCTATCGGG	GATCAAGTTAGACACTCCAG	GAAGCTAGAAGGTCCACCGG
VCP	ENSG00000165280	AATAACCTTCGTGTACGCCT	GACACAGTGATCCACTGCGA	TTAGCTCTCACCTTTCCGGA
RPA2	ENSG00000117748	TATGGCAGCTCCTCATACGG	ACAGGTGGATTCGAAAGCTA	ACTAAGCAAAGCCAACAGCC
USP11	ENSG00000102226	AGAACGGACGGCGATGGCGA	TGGGAGGCATACGTGCAGGG	AGAGCTGCCCAACATCCAGA
AKAP12	ENSG00000131016	GAAGAAGGAGCTACTTCCGA	CAAGCTGGAAGCCCTACCGA	TCAAGCCGAAACCTTAGCTG
MLH1	ENSG00000076242	AGTGGTGAACCGCATCGCGG	TAAGGTCTATGCCCACCAGA	GGTTAATGATCCTTCTCCGG
CDKN1B	ENSG00000111276	AAGAGGTTCCTGCAGGCCGA	CAGGAACCTCTTCGGCCCGG	GGACCACGAAGAGTTAACCC
RAD51	ENSG00000051180	AGAAGCTGGATTCCATACTG	GTCGAGGTGAGCTTTCAGCC	TTGGTGGAATTCAGTTGCAG
GTF2H1	ENSG00000110768	GAGTCAAGTGATCAGTGCTG	ATTGGAAGCAGATGGCACAG	ATTTAACCATCACAGTGCCA
COPS5	ENSG00000121022	ACAGCAGCAAGAAATCCTGG	GATGGGTCTGATGCTAGGAA	CCAACAAGAACAATATCCGC
GPN1	ENSG00000198522	TCCAGGTTGATCACATACGG	TATGGACTTGGACCCAATGG	CATTGACCACAGCTTTGCAG
NOP58	ENSG00000055044	TTAGCATCAGCTACTGCCAG	AATGGATGGATTAATCCCTG	CAACTCAGAAAGCTTGGCAG
FEN1	ENSG00000168496	TGGCCAAACGCAGTGAGCGG	TCTGAGGAGCGAATCCGCAG	GAGCCGCCAAGGCAGCACCC
NOP56	ENSG00000101361	AATGCCAACGCCGTGTCTGA	GATTGGTGCCGCAATACAGG	CAGCTTGTAAAGCACAGCTG
ERCC1	ENSG00000012061	ATTACGTCGCCAAATTCCCA	GAAACCAGCGGACCTCCTGA	GAGGGACCTCATCCTCGTCG
ERCC2	ENSG00000104884	GCGGGAGCTCAAACGCACGC	TTATCGGCAGGCATATCCGC	AAGGAACAGGTGCTCACCTC
UBA2	ENSG00000126261	TAAGAGGAACATCAGCTGCC	TGGGCTGAAGTACAAAGTCA	GCAGGATGACCCATCTGCAA
FGD5	ENSG00000154783	AATCTGGCAGCTCTCCTCGG	TGAAGAGCAGAGAAGCTCGG	AAGGCAGAGACACATTGGCC
COPS7A	ENSG00000111652	GCTCCTAGCCAAGTCGGCCA	AAACACTGTGAGCAGCCGGA	AGGTTGACTACAGCATCGGG
PRPF19	ENSG00000110107	CAACAACCAGCCTCTCTCCG	GGCACAATGAGGCCAGCCTG	GAAGTCACTGCTGCCCGAGA
RFC3	ENSG00000133119	GGGACGGCTGGACTATCACA	GCAGCACAAGATCAATCTGC	GCAGGCTTCACACATAAGCA
NDRG1	ENSG00000104419	AGCCGCTCGCACACCAGCGA	CAGCCGCTCGCACACCAGCG	AAGCCGCTCCCACACCAGCG
GADD45B	ENSG00000099860	GATGCAGACGGTGACCGCCG	GGAGAGCCGGCCGAGACCCA	GAAGAGCCACGGCTTGGTGG
FAM104A	ENSG00000133193	GCTGCGAGAGCCAAGGCCCG	GTGGAAGTGGGCCTCCCTCA	GAGCAGTCCGCGCTGTGCCA
MECP2	ENSG00000169057	GGACACGGAAGCTTAAGCAA	AGAAGGGTCAGGCTCCGCCC	GAGCGGCACCACGAGACCCA
DDB2	ENSG00000134574	GGGGCGTAATACAATCTCGG	AGGACGATCGACGTGTTCGA	CCGAGATTGTATTACGCCCC
Non-coding		GCATCTTGTAAAGTGAGCAA	CCAATAGCTGAATCATGTCA	

Table S4. ICP-MS parameters for Agilent 7800®.

RF power:	1550 W
Cone material:	Nickel
Carrier gas:	1.07-1.09 L/min
Plasma gas:	15 L/min
Monitored isotopes:	^{185}Re, ^{195}Pt, ^{196}Pt
Integration time:	1 s
Number of sweeps:	100
Number of replicates:	10
